# Reliability of trans‐generational genetic mark–recapture (tGMR) for enumerating Pacific salmon

**DOI:** 10.1111/eva.13647

**Published:** 2024-02-07

**Authors:** Samuel W. Rosenbaum, Samuel A. May, Kyle R. Shedd, Curry J. Cunningham, Randy L. Peterson, Brian W. Elliot, Megan V. McPhee

**Affiliations:** ^1^ College of Fisheries and Ocean Sciences University of Alaska Fairbanks Juneau Alaska USA; ^2^ Alaska Department of Fish & Game Anchorage Alaska USA; ^3^ Alaska Department of Fish & Game Douglas Alaska USA; ^4^ Alaska Department of Fish & Game Haines Alaska USA

**Keywords:** abundance estimation, close‐kin mark–recapture, dropout, individual‐based model, Pacific salmon, trans‐generational genetic mark–recapture, tGMR

## Abstract

As Pacific salmon (*Oncorhynchus* spp.) decline across much of their range, it is imperative to further develop minimally invasive tools to quantify population abundance. One such advancement, trans‐generational genetic mark–recapture (tGMR), uses parentage analysis to estimate the size of wild populations. Our study examined the precision and accuracy of tGMR through a comparison to a traditional mark–recapture estimate for Chilkat River Chinook salmon (*O. tshawytscha*) in Southeast Alaska. We examined how adult sampling location and timing impact tGMR by comparing estimates derived using samples collected in the lower river mainstem to those using samples obtained in upriver spawning tributaries. Results indicated that tGMR estimates using a representative sample of mainstem adults were most concordant with, and 3% more precise than, the traditional mark–recapture estimate for this stock. Importantly, the timing and location of adult sampling were found to impact abundance estimates, depending on what proportion of the population dies or moves to unsampled areas between downriver and upriver sampling events. Additionally, we identified potential sources of bias in tGMR arising from violations of key assumptions using a novel individual‐based modeling framework, parameterized with empirical values from the Chilkat River. Simulations demonstrated that increased reproductive success and sampling selectivity of older, larger individuals, introduced negative bias into tGMR estimates. Our individual‐based model offers a customizable and accessible method to identify and quantify these biases in tGMR applications (https://github.com/swrosenbaum/tGMR_simulations). We underscore the critical role of system‐specific sampling design considerations in ensuring the precision and accuracy of tGMR projects. This study validates tGMR as a potentially useful tool for improved population enumeration in semelparous species.

## 1 INTRODUCTION

Anthropogenic pressures are driving declines in Pacific salmon (*Oncorhynchus* spp.) abundance across much of their range (Beamish, 2022; Riddell et al., 2022), negatively impacting salmon‐reliant ecosystems, cultures, and economies. Efforts to restore Pacific salmon populations require accurate and precise estimates of key demographic quantitates, such as the number of mature adults returning to spawn, commonly referred to as “escapement”. Salmon fisheries are often managed to meet escapement goals, determined by state and federal agencies (Clark et al., 2006). Improving the reliability and efficiency of salmon escapement enumeration methods will aid sustainable management of Pacific salmon populations.

Mark–recapture experiments, which are commonly used for fishery management (Adkison, 2022), traditionally rely on physically tagging individuals for population abundance estimation (Pradel, 1996). While these methods can provide reliable abundance estimates under specific conditions, meeting necessary assumptions is challenging, often resulting in reduced precision of abundance estimates (Roff, 1973). Notably, physical tags may be lost or affect individual behavior, leading to potentially misleading estimates (Seber & Felton, 1981). Failure to identify recaptured individuals can lead to an over‐estimation of abundance, further imperiling declining populations by enabling over‐harvest. Innovations in tagging methodology are therefore beneficial for remedying these deficiencies.

Recent years have seen a growing interest in molecular applications of the mark–recapture framework, broadly known as close‐kin mark–recapture (CKMR) (Bravington et al., 2016). CKMR uses multi‐locus genotypes to mark individuals, and subsequent sampling of the close kin of marked individuals are treated as “recapture” events. Because the CKMR framework does not require physical tags or repeated sampling of individuals, it reduces sampling invasiveness while increasing efficiency. CKMR estimators frequently produce population estimates with higher precision than conventional abundance monitoring techniques (e.g., traditional mark–recapture and redd count expansion) for a broad range of iteroparous marine (Bravington et al., 2016; Delaval et al., 2023; Hillary et al., 2018; Patterson et al., 2022) and freshwater (Marcy‐Quay et al., 2020; Prystupa et al., 2021; Ruzzante et al., 2019; Wacker et al., 2021) fishes.

Trans‐generational genetic mark–recapture (tGMR) is a specific application of CKMR designed for enumerating semelparous species, such as Pacific salmon. Within the tGMR framework, the initial sampling event comprises genetic sampling of spawning adults, and the second sampling event involves genetic sampling of potential offspring (Rawding et al., 2014). A distinct advantage of tGMR lies in its ability to “tag” numerous offspring by genotyping only a comparatively small number of adults, thereby increasing precision through a greater number of recaptures. However, robust inference from mark–recapture estimates is contingent upon meeting or accounting for model assumptions (Seber, 1982). While tGMR and traditional mark–recapture assumptions are nearly identical (Peterson et al., 2023), methods for evaluating tGMR assumptions are not well‐developed, given that the availability of offspring for recapture is mediated by the reproductive success of adults. Specifically, the assumptions of (1) equal probability of capture within the first sampling event and (2) a closed population between sampling events may be violated when enumerating Pacific salmon (Rawding et al., 2014; Small et al., 2020) because sampling selectivity of adults may covary with reproductive success.

tGMR estimates could be biased if there are conditions that jointly influence the probability of certain adults and their offspring being captured (i.e., violating assumption 1). There are several aspects of Pacific salmon life history and common sampling techniques that could lead to these biases. For example, age, body size, and sampling selectivity are correlated in most salmonids and are often important determinants of reproductive success (reviewed in Koch & Narum, 2021). Thus, it is important to evaluate potential biases in tGMR estimates by examining the influence of age and size on variability in reproductive success and sampling selectivity.

Another factor complicating tGMR estimation is the biological complexity of freshwater migration and the potential for loss of adults between sampling events, violating assumption 2. Chinook salmon handled in mark–recapture or telemetry studies may enter rivers but later move back into the saltwater habitat, becoming vulnerable to marine sources of mortality including harvest (Sethi & Tanner, 2014). Additionally, prespawn mortality of adult salmon can occur within freshwater habitats (Bowerman et al., 2016), and mature adults can migrate to unsampled spawning habitat within a watershed. Past tGMR studies were limited to collecting adult tissue samples (“marks”) from carcasses only encountered in the upstream tributary habitat (Rawding et al., 2014; Small et al., 2020; Whitmore, 2016). However, by sampling mature adults both initially in the lower mainstem river and later in the upriver tributary habitats, separate tGMR estimates can be calculated, potentially allowing one to estimate rates of loss between mainstem and spawning reaches.

Individual‐based models are a powerful tool for examining population dynamics of Pacific salmon (Lin et al., 2017; May et al., 2023; Reed et al., 2011; Yeakel et al., 2018). Simulation models parameterized with empirical data have proved particularly useful for exploring emergent properties of life‐history dynamics that are difficult to measure in natural systems (Berdahl et al., 2018). Recent modeling work has broadly highlighted the importance of accounting for variation in life‐history traits and experimental design when implementing CKMR (Waples & Feutry, 2022). Developing a flexible and user‐friendly simulation framework to quantify bias specifically associated with violations of tGMR assumptions could help guide future tGMR applications.

The present study aimed to evaluate the suitability of tGMR methods for escapement estimation by exploring how differences in adult sampling can lead to bias. To meet this goal, we conducted a case study on Chinook salmon (*O. tshawytscha*) returning to the Chilkat River, Alaska, in 2020 with the following objectives: (1) compare abundance estimates and their precision derived from tGMR with traditional mark–recapture; (2) investigate the effect of variation in reproductive success and adult sampling selectivity on tGMR abundance estimates (assumption 1 violations); and (3) explore the effect of adult sampling location and timing on tGMR abundance estimates (assumption 2 violations). To achieve these objectives, we calculated tGMR abundance estimates using adults sampled in both the lower mainstem river during initial freshwater migration and later in upriver tributaries during spawning. We then designed an individual‐based simulation framework for examining sources of bias in tGMR applications, which can be used in other semelparous species and systems to evaluate the impact of assumption violations (https://github.com/swrosenbaum/tGMR_simulations). tGMR may provide a more efficient and less invasive tool to enumerate adult salmon populations and could offer increased precision when compared with traditional mark–recapture methods. By addressing key knowledge gaps, we aimed to assess the reliability of extending tGMR across the range of semelparous salmon for improved enumeration of these iconic species.

## 2 METHODS

### 2.1 Study system

**FIGURE 1:**
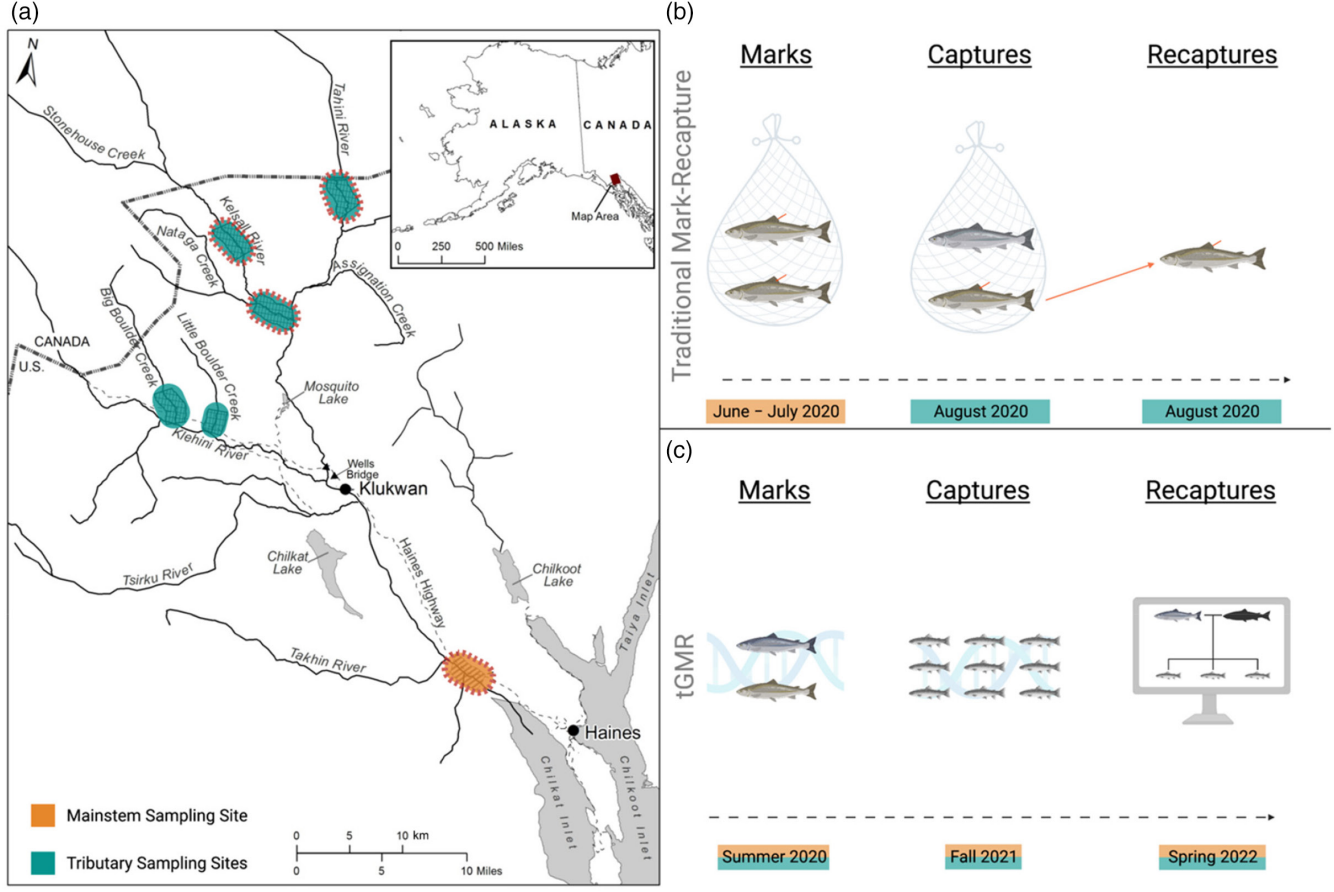
(a) Map of the Chilkat River watershed near Haines, AK. Chinook salmon were sampled in the mainstem (orange) and tributaries (blue). Sampling areas with a dashed red border denote locations where both adult and juvenile Chinook salmon were collected. Areas without the dashed red border indcate that only adults were sampled. (b) Diagram of a traditional mark–recapture program used to estimate the escapement of Chilkat River Chinook salmon in 2020. (c) Diagram of the trans‐generational genetic mark–recapture (tGMR) framework used to estimate escapement of Chilkat River Chinook salmon in 2020, created with BioRender.com.

In 2020, a tGMR experiment was conducted on Chinook salmon returning to the Chilkat River, located near Haines, AK (Figure 1a). The Chilkat River is the third largest producer of Chinook salmon in Southeast Alaska (McPherson et al., 2003) and is an exploitation rate and escapement indicator stock monitored by the Pacific Salmon Commission under the Pacific Salmon Treaty (Chinook Technical Committee, 2023). The Chilkat River Chinook salmon stock is critical to subsistence fishers in the region, who catch as much as 17% of the total Chilkat River Chinook salmon harvest in some years. The Chilkat River Chinook salmon stock only reached its escapement goal once during 2012–2017, resulting in its designation as a “stock of management concern” by the Alaska Department of Fish and Game (ADF&G) in 2017 (Lum & Fair, 2018). Following this designation, the estimated 2018 escapement was the lowest in the history of the time series. Due to this population's decline and subsequent ADF&G management designation, the subsistence fishery for Chilkat River Chinook salmon has been closed since 2017 (Elliott & Peterson, 2022). In an era of declining salmon populations and decreasing budgets for management agencies, fewer resources are available for monitoring activities, and managers may implement precautionary approaches by further reducing the harvest.

The Chilkat River Chinook salmon stock is currently monitored through a long‐term traditional mark–recapture project occurring annually since 1991 (Ericksen & McPherson, 2004), making it an excellent system in which to evaluate tGMR methods. The existing traditional mark–recapture project utilizes multiple gears to capture fish in both the lower mainstem river during freshwater migration (adult event 1; fishwheels and drift gillnets) and upriver tributary sampling on spawning grounds (adult event 2; rod and reel, seining, and/or carcass sampling). The combination of sampling gear and their associated biases is discussed below and has been implemented by ADF&G to estimate the abundance of large adult Chinook salmon ≥ age 4 (1.2 in European notation, Koo, 1955). The successful application of tGMR for estimating the escapement of Chilkat River Chinook salmon may allow extension of this method to other Alaskan salmon stocks that currently lack intensive monitoring programs for more precise escapement estimation.

### 2.2 Sampling

Elliott and Peterson (2022) estimated the abundance of returning Chinook salmon (≥ age 4; 1.2 in European notation) entering the Chilkat River in 2020 using a traditional two‐sample mark–recapture study for a closed population (Seber, 1982). Returning adult Chinook salmon were captured during freshwater migration in June and July using fishwheels and drift gillnets (7 ½ inch mesh) during event 1 in the lower mainstem of the Chilkat River and marked with Floy T‐bar tags (Figure 1b). Use of fishwheels (selective for smaller, younger fish) and drift gillnets (selective for larger, older fish) helped ensure that a representative sample of all adult size and age classes was collected (Elliott, 2022). Event 2 sampling occurred during August in the three major spawning tributaries of the upper Chilkat River using rod and reel, dip nets, short tangle nets, beach seines, and carcass surveys. Potential biases and sources of errors were evaluated using the methods outlined in Elliott (2022). Abundance of adult Chinook salmon (excluding age‐3 males, 1.1, or “jacks”) was estimated using Chapman's modification of Petersen's mark–recapture method (Chapman, 1951). Jacks were excluded from the traditional estimate as the capture rate for these younger, smaller individuals on the spawning grounds during the adult event 2 sampling was insufficient for robust estimation of this age‐class (proportion sampled = 0.01). We used the results of this traditional mark–recapture effort to compare the precision and agreement of our tGMR case study detailed below.

Tissue samples were collected from the adults sampled as part of the traditional mark–recapture study (adult events 1 and 2; Elliott, 2022). Both events were treated as ‘marks’ for the tGMR method. Pelvic fin tissue samples were collected and dried on Whatman paper to preserve DNA. Additional metadata were recorded for each individual, including body length (mid eye to tail fork), sex, sample date, sample location, and age from scale samples (Peterson et al., 2023).

Juvenile tissue samples were collected in the fall of 2021 from fall parr rearing in the Chilkat River using the methods described in Elliott and Peterson (2022). These samples represent our second sampling event in the tGMR experiment (hereafter ‘captures’). Parr were sampled using a stratified systematic sampling design, with samples collected continuously throughout September and October, across the mainstem and two of the three primary spawning tributaries of the Chilkat River using baited minnow traps (Figure 1c). As an alternative to fin clipping parr Chinook salmon, tissue samples were collected non‐lethally using OmniSwabs (Qiagen, Whatman FTA), which were used to collect DNA from external fish mucus in a minimally invasive manner. OmniSwabs were preserved dry in 2‐mL cryovials filled with silica desiccant beads to preserve DNA. We recorded additional metadata for each individual, including body length (fork length), sample date, and sample location.

### 2.3 Molecular protocol

Genetic analysis was conducted at the ADF&G Gene Conservation Laboratory in Anchorage, AK. Following the methods outlined in Peterson et al. (2023), we extracted genomic DNA from adult pelvic fin samples and parr OmniSwabs swabs separately using NucleoSpin® Tissue Kits (Macherey–Nagel). We genotyped each sample using two different methods: (1) Genotyping‐in‐Thousands by sequencing (GT‐seq; Campbell et al., 2015) for 299 single‐nucleotide polymorphism (SNP) genetic markers in the 299 SNP v3.0 GT‐seq panel developed by the Columbia River Inter‐tribal Fish Commission Hagerman Genetics Laboratory (Hess et al., 2014; Appendix S1) and (2) polymerase chain reaction (PCR) fragment analysis for five multi‐plexed microsatellite loci from the Genetic Analysis of Pacific Salmon (GAPS) panel (Moran et al., 2013; Seeb et al., 2007; Appendix S1).

GT‐seq SNP libraries were sequenced on an Illumina NextSeq 500 with single‐end 150 base‐pair reads. Individual genotypes were called using *GTscore* (https://github.com/gjmckinney/GTscore), a custom GT‐seq genotype‐calling pipeline that uses sequence matching to quantify allelic count and ratios for each marker to infer the genotype (McKinney et al., 2020). We then imported our genotype data into the ADF&G database LOKI. SNPs in the GT‐seq panel were removed from use in downstream analyses based on their performance in the Southeast Alaska Chinook salmon genetic baseline (Shedd & Gilk‐Baumer, 2021). Briefly, Shedd and Gilk‐Baumer (2021) excluded loci if (1) visual examination of allelic ratio plots indicated non‐singleton allelic ratios (i.e., diverged duplicate or duplicated loci), (2) loci failed to conform to Hardy–Weinberg expectations (HWE), and (3) if pairs of loci were in linkage disequilibrium (LD).

Five of thirteen microsatellite loci from the GAPS panel (*Omm1080*, *Ots213*, *Ots201b*, *Ssa408uos*, and *Ots9*) were amplified in a single, multiplexed PCR reaction following methods described in Seeb et al. (2007). PCR products were visualized using a 3730 capillary DNA analyzer (Applied Biosystems). Genotypes were scored manually with GeneMapper software (version 4.0, Applied Biosystems) and then imported into the ADF&G database LOKI.

To identify laboratory errors and quantify the genotyping error rate for use in downstream parentage analyses, 8% of sampled individuals were re‐extracted and assayed for the same set of markers described above. The discrepancy rate, which reflects DNA extraction, assay plate, and genotyping errors, was calculated as the number of conflicting genotypes divided by the total number of genotypes compared. The discrepancy rate was then divided by 2 to give the genotyping error rate.

Following procedures described in Shedd et al. (2022), we adapted custom scripts (https://github.com/krshedd/GCL‐R‐Scripts) to further filter our genotype data using the programming language R (R Core Team, 2023). These scripts removed individuals missing 20% or more of their genotypes, duplicate individuals identified as sharing at least 95% of their genotypes, and potentially contaminated individuals identified by excessively heterozygous SNP genotypes (defined by a cutoff of 1.5 times the interquartile range). These steps were intended to reduce quality issues resulting from low‐grade DNA, duplicate sampling, and contamination.

### 2.4 tGMR abundance estimation

To determine kinship relationships among adults and juveniles, the parentage analysis program COLONY was used to reconstruct a one‐generation pedigree (version 2.0.6.8) (Wang & Santure, 2009). COLONY is a full probability pedigree reconstruction software that uses maximum likelihood to reconstruct full‐ and half‐sibling family groups among juveniles and assigns parents to family groups. Additionally, COLONY infers genotypes of unsampled parents through information of sibling relationships among juveniles. Reconstructing unsampled parental genotypes allows for putative identification of the total number of reproductively successful adults, both sampled and unsampled. Simulations indicated a large number of polymorphic markers (several hundred) are necessary for valid inference of unsampled parents when using prior versions of COLONY (version 2.0.6.1) (Whitmore, 2016). These inferred parental quantities are necessary for the hypergeometric implementation of tGMR (described below). We decided to not use sex data as an input to COLONY as non‐lethal identification of sex in adult salmon is error‐prone (Chapell, 2014), especially in the lower river mainstem, and the genotyping panel used in these analyses lacks a reliable sex marker. We assumed male and female polygamy without inbreeding. All COLONY input parameters are provided in Table S1. To assess the potential influence of *en route* loss of adults (from here on referred to as “dropout”) on tGMR abundance estimates, we ran COLONY on three separate datasets to identify pedigree relationships between (1) all adults and all juveniles; (2) adults sampled in the lower mainstem river and all juveniles; and (3) adults sampled in the upriver tributaries and all juveniles. Prior to each of the three COLONY runs, we identified and removed any duplicate individuals (including adult recaptures when analyzing all adults) (Table S2).

Abundance estimates were derived from pedigree outputs from COLONY using both the binomial and hypergeometric tGMR models described by Rawding et al. (2014) and later adjusted by Small et al. (2020) and Peterson et al. (2023). Briefly, the binomial estimator allows sampling with replacement and estimates the adult population size (*N*_bin_) from the number of adults genotyped (marks, *M*), the number of parr genotyped multiplied by 2 (captures, *C*), and the number of POPs (recaptures, *R*). The number of sampled parr is multiplied by 2 because the parental genotype is the mark, and because juveniles carry genotypes from dams and sires, they have two opportunities to recapture marks. Under this sampling‐with‐replacement framework, all juvenile data are incorporated into the estimator regardless of whether multiple juveniles share one or both parents (resampling). In other words, siblings from the same parent are counted as separate recaptures and represent unique POPs. On the other hand, the hypergeometric estimator implements sampling without replacement, where only the first ‘sampling’ of a parent by a genotyped parr is considered. This estimator relies on the number of unsampled parents inferred by COLONY. In this case, *M* is still the number of adults genotyped, but *C* is the number of unique parents inferred from offspring kinship relationships, both sampled and unsampled, and *R* is the number of unique sampled parents assigned to at least one offspring.

Escapement estimated using Bailey's modified binomial model (Small et al., 2020) was

(1) $$N_{\mathrm{bin}}=\frac{M\left(C+1\right)}{\left(R+1\right)}$$

Variance was estimated using Bailey's (1951) approximation:

(2) $$\mathrm{var}\left(N_{\mathrm{bin}}\right)=\frac{M^{2}\left(C+1\right)\left(C-R\right)}{{\left(R+1\right)}^{2}\left(R+2\right)}$$

Escapement estimated using Chapman's modified hypergeometric model was

(3) $$N_{\mathrm{hyp}}=\frac{\left(M+1\right)\left(C+1\right)}{\left(R+1\right)}-1$$

Variance of *N*_hyp_ was estimated as

(4) $$\mathrm{var}\left(N_{\mathrm{hyp}}\right)=\frac{\left(M+1\right)\left(C+1\right)\left(M-C\right)\left(C-R\right)}{{\left(R+1\right)}^{2}\left(R+2\right)\;}$$

Confidence intervals for both $N_{\mathrm{bin}}$ and $N_{\mathrm{hyp}}$ were calculated using a normal approximation as the number of recaptures was expected to be large.

### 2.5 Simulations to quantify bias from assumption violations

#### 2.5.1 Individual‐based model overview

**FIGURE 2:**
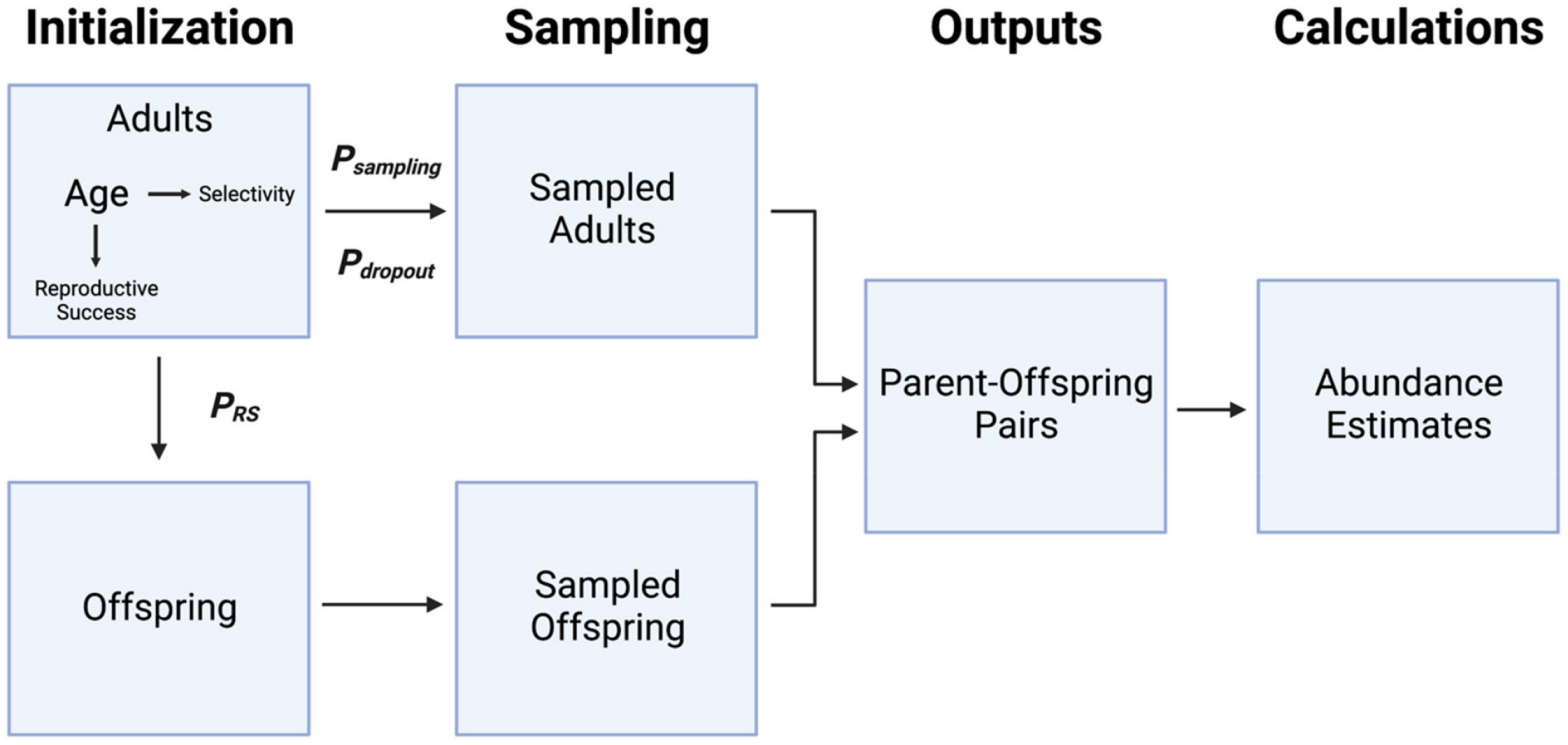
Schematic of our individual‐based model workflow to quantify the reliability of trans‐generational genetic mark–recapture (tGMR) for Chilkat River Chinook salmon under varying demographic and sampling scenarios. Created with BioRender.com.

To examine the accuracy and precision of tGMR estimation under varying demographic and sampling scenarios, we implemented a paired simulation–estimation approach. Specifically, we constructed an individual‐based simulation model of a single semelparous salmonid population across one generation, in which adult salmon were sampled as they returned to spawning grounds (marks), and later their juvenile offspring were sampled (captures; Figure 2). This model was used to examine the effect of two variables on tGMR abundance estimation: (1) age‐specific differences in adult reproductive success (**P**_**RS**_) and (2) age‐specific differences in adult sampling selectivity (**P**_**sampling**_), which were both vectors of age‐specific probabilities input as initial parameters. Age‐specific differences in reproductive success were defined as the relative probability of individuals of a certain age producing at least one offspring when compared to a different age class. Age‐specific differences in adult sampling selectivity were defined as the relative probability of individuals of a certain age class being sampled during event 1 of tGMR sampling as they returned to spawning grounds. These demographic parameters are expected to bias tGMR estimates through their combined effect on the number of recaptures identified through parentage analysis (Waples & Feutry, 2022). We parameterized the model with values from our empirical tGMR case study on Chinook salmon from the Chilkat River to quantify the direction and magnitude of bias in tGMR estimates arising from differences in age‐specific reproductive success and non‐uniform event 1 (adult) sampling selectivity at age.

### 2.6 Model initialization

We initialized the simulation with a known number of adults (Nc_adults_) and offspring (Nc_offspring_) parameterized based on our case study system (Table 1). Each adult was assigned three parameter values: (1) age, (2) probability of reproductive success, and (3) probability of being sampled conditional on the assumed selectivity of the event 1 adult sampling process. First, ages (**Ages**) were assigned to individuals using a weighted draw from a vector of ages (3, 4, 5, or 6 years old) proportional to the observed average age composition of Chilkat River adult Chinook salmon across the mainstem 2020 sampling effort (**P**_**age**_). This initial input parameter determined the age structure of the returning adult population encountered during event 1 sampling. Second, reproductive success probabilities for individual adults were dependent on their age and were assigned from a constant vector of age‐specific reproductive success probabilities (**P**_**RS**_), such that all individuals of a given age had the same probability of having offspring. There was no additional random variation in age‐specific reproductive success values, although such variation could be incorporated in future model applications. **P**_**RS**_ was estimated from empirical data from both mainstem and tributary adults as a vector of age‐specific mean reproductive success values, where reproductive success was a Bernoulli variable (zero offspring or >0 offspring). Third, individual sampling probabilities were also dependent on age values and assigned from a vector of age‐specific sampling selectivity values (**P**_**sampling**_). A subset of parents in the simulated spawning population were sampled (*n*_adults_) as they returned to hypothetical natal rivers, conditional on their age‐specific sampling probability. Parents were assigned to offspring using a weighted draw of *n*_adults_ from all possible parents in the population (Nc_adults_). We did not parameterize these values differently by sex, but this could be implemented in future applications.

**TABLE 1:** Input paramters and example values for individual‐based simulations of Chilkat River Chinook salmon.

Argument	Definition	Example values
Nc_adults_	Adult census size	3702
Nc_offspring_	Offspring census size	480,000
*n*_adults_	Adult sample size	581
*n*_offspring_	Offspring sample size	682
**Ages**	Adult ages	3, 4, 5, 6
**P**_**age**_	Adult age structure probability	0.1, 0.14, 0.64, 0.12
**P**_**RS**_	Age‐specific reproductive success probability	0.07, 0.17, 0.28, 0.38
**P**_**sampling**_	Age‐specific sampling probability	0.03, 0.30, 0.38, 0.29
P_dropout_	Age‐specific probability of removal of individuals from the population between mainstem and tributary sampling events	0.05
Proportion_dropout_	Proportion of adults that are randomly removed from the population between simulated mainstem and tributary sampling events	0.30
Iterations	Number of iterations performed for each unique combination of input parameters	1000
Scenario	Scenario identifier	1
δ_**RS**_	Slope of a logistic function predicting age‐specific probabilities of having offspring	−3, −2, −1, 0, 1, 2, 3
δ_**sampling**_	Slope of a logistic function predicting age‐specific probabilities of being sampled	−3, −2, −1, 0, 1, 2, 3

The value *N*_offspring_ determined the census size of parr in the next generation, which was held constant across simulations in this study. Offspring were each assigned one parent, using methods adapted from *The Weight* (Waples, 2022) by using a random draw from all possible parents, weighted by **P**_**RS**_ values of parents. Then, a proportion of offspring were randomly sampled from all offspring in the population, representing juvenile sampling. The number of offspring sampled was also set as an initial input parameter (*n*_offspring_). We performed a hypothetical molecular pedigree reconstruction from sampled parents and offspring, assuming perfect parentage assignment. For each unique set of input parameters, the simulation was re‐run for 1000 iterations to quantify uncertainty.

### 2.7 Model outputs

Model outputs comprised an incomplete pedigree containing sampled offspring and their assigned parent, if sampled, in addition to the ages of the known parents. These data were then used to quantify key population abundance statistics, such as the number of POPs and a tGMR estimate analogous to a single‐sex binomial estimate. Mean tGMR estimates (Equation 5) and the range of abundances predicted by 95% of the simulations were quantified across 1000 iterations per simulation. By not accounting for sex, our model produces tGMR estimates that do not directly follow the currently established binomial or hypergeometric tGMR frameworks. Our simplified approach assumed equal sex ratios and no variability in reproductive success or sampling selectivity between the sexes.

(5) $$\text{Averaged Model Estimate}=\frac{\left(\text{mean}\left(n_{\text{adult}}\right)\times \left(\text{mean}\left(n_{\text{offspring}}\right)\right)+1)\right)}{\left(\text{mean}\left(\text{POPs}\right)+1\right)}$$

### 2.8 Model parameterization

**P**_**RS**_ was calculated from our empirical Chilkat River case study by identifying the mean reproductive success for each age‐class. We used 434 ages from scale samples collected from both the lower river and the upstream tributaries from the case study system, and we used only these 434 individuals with known ages for reproductive success analysis (Figure S1).

**P**_**sampling**_ was also calculated from the case study data using the 434 adults with known ages from scale age analysis. We first estimated the “true” age structure of the population using adults sampled from the lower mainstem. As discussed previously, adults sampled in the lower mainstem river are thought to provide the most accurate representation of the population's age structure due to the use of fishwheels and gillnets with differing selectivity. Next, the relative selectivity for the upriver tributary sampling event was estimated by calculating the age structure within tributary adults by dividing the number of adults of a given age within the tributary by the “true” age structure calculated from the mainstem adults. Finally, we standardized selectivity within the tributary by dividing the relative age structure for the tributary sampling event by the sum of the relative age structure for the tributary sampling event.

### 2.9 Asymptotic reproductive success and sampling selectivity (violating assumption 1)

To examine how age‐specific differences in reproductive success and sampling selectivity influence the accuracy and precision of tGMR estimation, we parameterized our model with a range of values for the age‐specific vectors **P**_**RS**_ and **P**_**sampling**_. To explore a range of possible scenarios in which reproductive success or selectivity of adult sampling varied with age, we generated values for these parameters from a logistic function (Equation 6), where the slope of the relationship between reproductive success or sampling selectivity and age was determined by a slope variable (δ), and the a50 parameter was fixed at the mean of observed ages (4.5).

(6) $$P_{\mathrm{RS}}\;\text{or}\;P_{\text{sampling}}\;\text{for}\;a\;\text{given}\;\delta \;\text{scenario}=1+e^{{\left(-1*\delta \left(\mathrm{age}-a50\right)\right)}^{-1}}$$

We scaled δ continuously across the values −3 to 3, which encompasses a range of plausible life history patterns of these traits for many salmonids and drew **P**_**sampling**_ and **P**_**RS**_ values across the scope of δ values. For each δ value, the corresponding **P**_**RS**_ and **P**_**sampling**_ vectors were used as simulation inputs while holding all other input parameters constant. **P**_**RS**_ and **P**_**sampling**_ were examined separately, varying only one parameter at a time while holding the other constant at values of 0.07, 0.17, 0.28, and 0.38 for **P**_**RS**_ and 0.10, 0.40, 0.70, or 0.10 for **P**_**sampling**_. The constant values reflect estimated quantities from the combined mainstem and tributary Chilkat River adult sampling events. Each δ scenario was evaluated across 1000 model iterations for each set of **P**_**RS**_ and **P**_**sampling**_ values separately, with among‐iteration differences arising from random variation in which: (1) individual adults were captured during event 1; (2) individual adults produced offspring; and (3) individual offspring were sampled during event 2. The model outputs from these iterations quantified the range of bias in tGMR estimates resulting from variation in reproductive success‐at‐age and selectivity‐at‐age predictions. Bias was calculated as the difference between the averaged point estimate across the 1000 iterations and the “true” input Nc_adults_ value, divided by Nc_adults_ (Equation 7).

(7) $$\text{Bias}=\frac{\left(\text{Averaged Model Estimate}-Nc_{\text{adults}}\right)}{Nc_{\text{adults}}}$$

### 2.10 Comparison of mainstem vs tributary tGMR estimates (violating assumption 2)

To examine how adult sampling location and timing of capture may impact tGMR estimates, we compared hypergeometric tGMR estimates inferred based on three adult Chinook collections: (1) lower mainstem Chilkat River adults sampled in June and July; (2) upriver tributary adults sampled in August; and (3) adults from both sampling events, combined. We used separately reconstructed pedigrees for these three collections. The resulting hypergeometric tGMR estimates and their confidence intervals (calculated using a normal approximation) were then compared. Hypergeometric estimates were used as opposed to binomial estimates because the hypergeometric model is more robust to violations of assuming an equal probability of capture during the adult sampling event (Rawding et al., 2014; Small et al., 2020). The hypergeometric model is generally more robust in salmonid systems, as the sampling without replacement framework buffers against heterogeneity in reproductive success, which may lead to violations of this core assumption (Small et al., 2020).

Differences in tGMR estimates between lower mainstem and upriver tributary samples could result from violations of assumptions in either estimate or the loss of adults from the population during freshwater migration. We defined dropout as the number of adults that may be sampled in the lower mainstem river but are subsequently unable to be sampled in the upriver tributaries for any reason that would violate the assumption of a closed population, such as movement out of the study area or accidental capture mortality. The potential influence of adult dropout on tGMR abundance estimation was examined with our individual‐based modeling framework, which assumes that assumptions are otherwise met for both estimates. Nine simulation scenarios were implemented with varying rates of dropout from 0% to 40%, in increments of 5%, based on a range thought to encompass a plausible set of values likely to occur in Alaska watersheds (Richards et al., 2017). Input **P**_**sampling**_ values for the lower mainstem and upriver tributary simulation scenarios were re‐calculated separately for each traditional adult sampling event/gear type in the same manner described in the “Model Parameterization” section. These re‐calculations were performed to better reflect the selectivity conditions occurring during these discrete sampling periods (Table 2; Figure S2), improving our ability to compare simulated and empirical results. Model outputs (e.g., mean tGMR estimates) were then compared to the empirical hypergeometric mainstem and tributary tGMR estimates.

**TABLE 2:** Age structure and selectivity for adult Chinook salmon, separated by adult age‐class, across lower mainstem and upriver tributary sampling efforts.

Parameter	Age 3	Age 4	Age 5	Age 6
Mainstem Age Structure	18%	12%	60%	10%
Mainstem Selectivity	18%	12%	60%	10%
Tributary Age Structure	1%	16%	69%	14%
Tributary Selectivity	1%	34%	29%	36%

## 3 RESULTS

### 3.1 Genotyping

The average genotyping success rate across all adult fish using GT‐seq was 99.70%. The average genotyping success rate across all juveniles using GT‐seq was 98.23%. There were 41 of the 641 adults and seven of the 700 parr that had contamination scores deemed too high to allow for accurate microsatellite genotyping. The entire multiplex of five microsatellite loci were retained for analyses; however, 26 of the 299 SNPs were dropped due to sequencing issues (non‐singleton allelic ratios), 11 SNPs were dropped due to violations of HWE, and eight SNPs were dropped due to LD with other SNPs (Shedd & Gilk‐Baumer, 2021). Our final genotyping panel consisted of 259 loci (254 SNPs and five microsatellites).

We removed 10 individuals from analyses who were missing greater than or equal to 20% of their genotypes (Dann et al., 2012). Furthermore, we removed 20 duplicated fish from our study. Most of these duplicates were recaptured adults that had been sampled during both adult sampling occasions (mainstem and tributary projects). We removed 46 outliers that fell outside the 1.5 interquartile range of individual heterozygosity. These individuals were likely excessively heterozygous because of contamination. The final dataset considered in all subsequent analyses (Table S2) included 583 adults (sample locations described in Table S3) and 682 juvenile Chinook salmon (sample locations described in Table S4).

### 3.2 Traditional and tGMR abundance estimates

**FIGURE 3:**
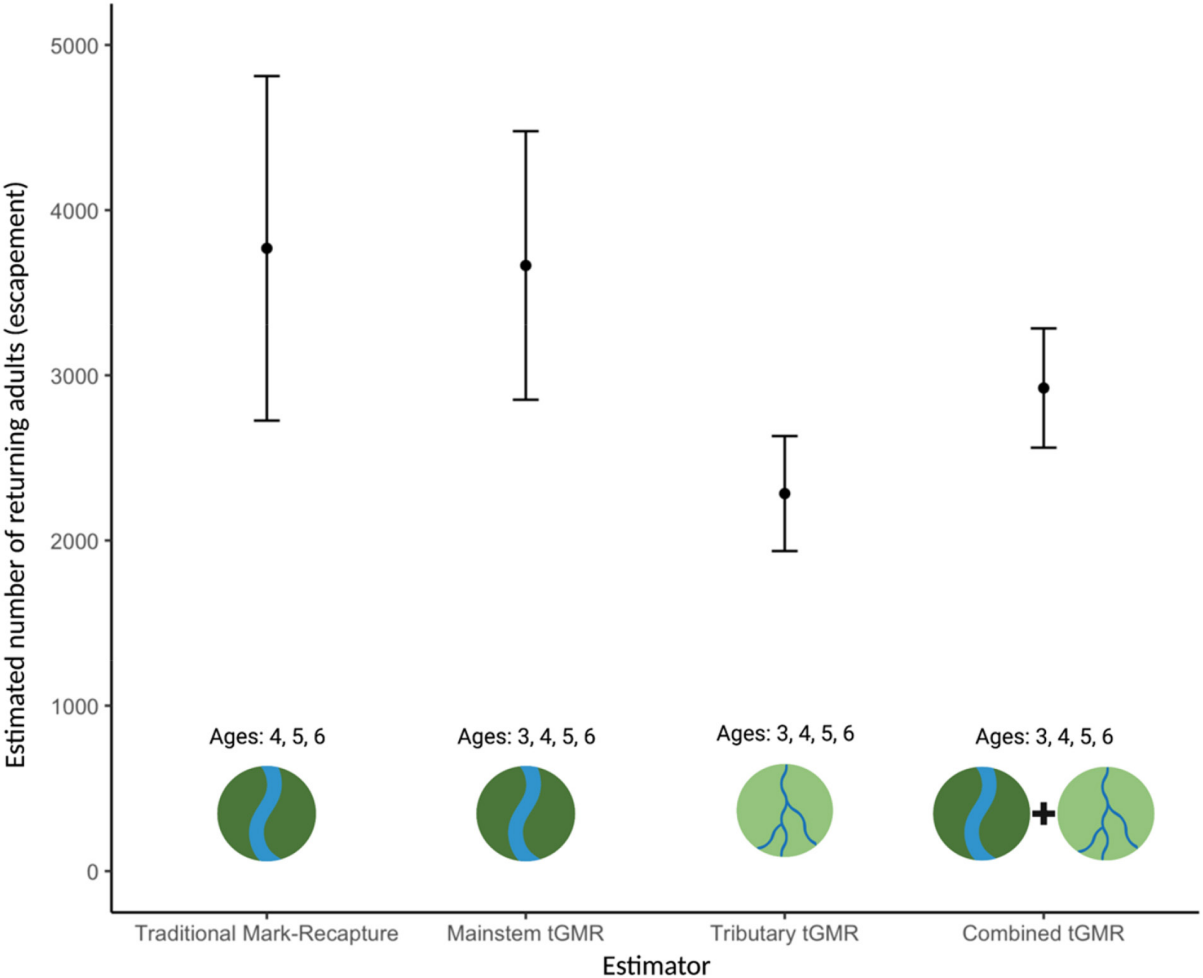
Estimated number of returning adults (escapement; *y*‐axis) from four different estimation methods (*x*‐axis) of adult Chilkat River Chinook salmon. Estimation methods include a traditional mark–recapture survey (left), which we compare to three hypergeometric tGMR estimates (right), quantified using samples collected on the river mainstem, tributaries, or both. Estimates are bounded by 95% confidence intervals. Icons created with BioRender.com.

Elliott and Peterson (2022) estimated the 2020 escapement of non‐jack Chilkat River Chinook salmon (≥age 4) to be 3769 fish (95% CI: 2726 – 4812; Figure 3; Table 3).

**TABLE 3:** Comparison of traditional and trans‐generational genetic mark–recapture (tGMR) estimators for Chilkat River Chinook salmon.

Estimator	*M*	*C*	*R*	Estimate	95% CI	CV (%)
Traditional Mark‐Recapture (adults ≥ age 4)	n/a	n/a	n/a	3769	2726–4812	14
Hypergeometric tGMR (all adults)	583	745	148	2923	2562–3284	6
Hypergeometric tGMR (mainstem adults)	295	705	56	3665	2852–4478	11
Hypergeometric tGMR (tributary adults)	306	721	96	2284	1936–2632	8
Binomial tGMR (all adults)	583	1364	237	3344	2958–3729	6
Binomial tGMR (mainstem adults)	295	1364	83	4794	3806–5781	11
Binomial tGMR (tributary adults)	306	1364	165	2516	2159–2874	7

*Note*: The traditional mark–recapture estimate is provisional, based on mark (*M*), capture (*C*), and recapture (*R*) values not currently being published (Elliott & Peterson, 2022).

COLONY identified 237 POPs, 148 unique sampled parents, and 745 total inferred parents from within the complete sample of 583 adults and 682 juvenile Chinook salmon. The 583 adult genotypes were considered the marks in both the binomial (with replacement) and hypergeometric (without replacement) estimators (Table 3). The captures in the binomial model were the 682 juveniles genotyped multiplied by 2 (each offspring has two potential parents), and the recaptures in the binomial model were the 237 POPs. The 745 unique parents inferred from the offspring using COLONY were considered the captures for the hypergeometric model, and the 148 unique sampled parents assigned to the offspring were the recaptures in the hypergeometric model. The escapement estimate calculated with the binomial model using all sampled adults was 3344 fish (95% CI: 2958 – 3729). Using the hypergeometric model while including all adults, we estimated the escapement to be 2923 spawners (95% CI: 2562–3284; Figure 3; Table 3).

### 3.3 Simulations to quantify bias from assumption violations

#### 3.3.1 Model parameterization

The mean probability of reproductive success at a given age (**P**_**RS**_) values was 0.07 for age−3 adults, 0.17 for age‐4 adults, 0.28 for age‐5 adults, and 0.38 for age‐6 adults (Figure S1). The calculated age structures and selectivity for each adult sampling event are presented in Table 2 and Figure S2.

#### 3.3.2 Asymptotic reproductive success (violating assumption 1)

**FIGURE 4:**
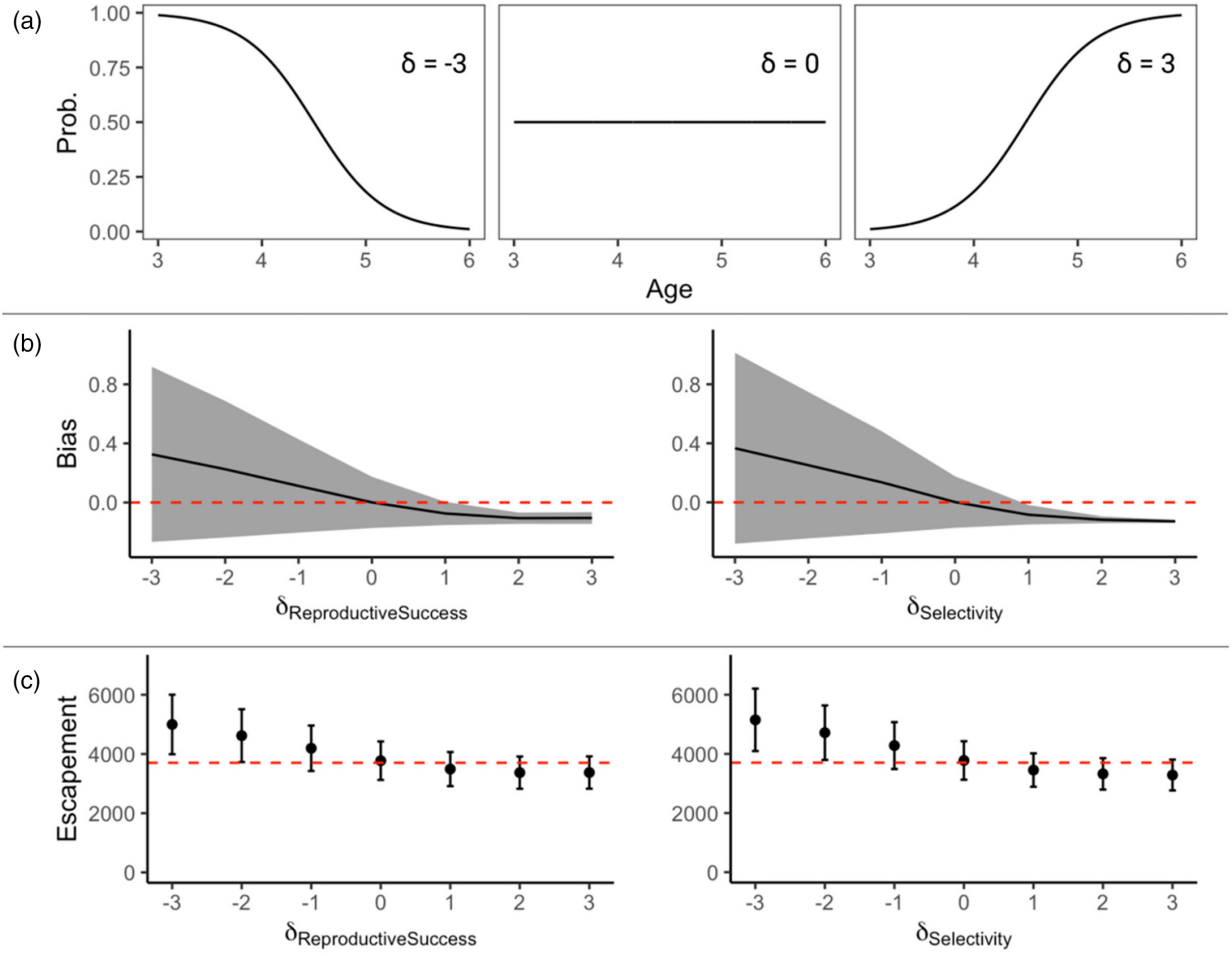
(a) Example scenarios varying the slope variable (δ) to quantify reproductive success and selectivity probabilities (*y*‐axis) for different ages (*x*‐axis) of Chilkat River Chinook salmon (Equation 7 in the text). (b) Expected bias (*y*‐axis) in trans‐generational genetic mark–recapture (tGMR) estimates resulting from age‐specific differrences (*x*‐axis) in reproductive success (left) and sampling selectivity (right). The shaded area indicates the range of bias predicted by 95% of simulations. The red dashed line represents zero bias. (c) Simulated average tGMR estimates, using a single‐sex binomial framework, and the 95% range of abundances predicted across 1000 iterations for each δ scenario. The red dashed line represents the true simulated population size.

We investigated how variation in age‐specific reproductive success influenced the magnitude and direction of bias in tGMR abundance estimation. Bias (i.e., the difference between model‐estimates and the true adult run size (Nc_adults_); Equation 7) increased as reproductive success‐at‐age decreased. For example, in simulations testing the most extreme decreasing age‐specific reproductive success (slope δ = −3), the averaged estimate was positively biased by 33% (Figure 4b). In contrast, bias was close to 0 (0.1%) when reproductive success was constant across ages (δ = 0). However, bias became more negative as age‐specific reproductive success increased. For example, in the most extreme case of increasing reproductive success‐at‐age (δ = 3), the averaged point estimate was biased by −10%. Notably, in scenarios with increasing reproductive success‐at‐age, bias appears to asymptote near −10%. The variability in bias among simulations decreased as δ became increasingly positive (i.e., greater reproductive success at older ages). In contrast, bias did not appear to asymptote with decreasing reproductive success‐at‐age. The range of bias observed in 95% of simulation iterations for a given δ is represented by the gray shaded area in Figure 4b. Chinook salmon reproductive success is positively associated with age (Koch & Narum, 2021), indicating that the scenarios simulating decreasing age‐specific reproductive success (δ = −3, δ = −2, δ = −1) are unlikely to occur in natural systems. However, we include these scenarios as some semelparous fishes may demonstrate this life history pattern, and it is therefore useful to fully understand the range of possible effects driven by variable age‐specific reproductive success in different systems.

#### 3.3.3 Asymptotic adult sampling selectivity (violating assumption 1)

We also investigated how variation in age‐specific sampling selectivity influenced the magnitude and direction of bias in tGMR abundance estimation (Figure 4). Bias increased as selectivity‐at‐age decreased. In simulations with the most extreme decreasing selectivity‐at‐age (δ = −3), the averaged point estimate was positively biased by 37%. However, bias was nearly 0 (0.2%) when selectivity was constant across ages (δ = 0). Bias became more negative as selectivity‐at‐age increased. Under the most extreme case of increasing selectivity‐at‐age (δ = 3), the averaged point estimate was biased by −13%. While bias appeared to increase severely and continuously with decreasing selectivity‐at‐age, under increasing selectivity‐at‐age, bias appeared to asymptote negatively at −13%. The variability in bias for δ = 3 decreased to nearly 0.

### 3.4 Comparison of mainstem vs tributary tGMR estimates (violating assumption 2)

Using 295 returning adult Chinook salmon sampled in the lower mainstem of the Chilkat River in June of 2020 and 682 parr collected in the fall of 2021, COLONY identified 56 unique adult parents assigned to parr offspring and inferred 705 total unique successfully reproducing adults. These quantities produced a hypergeometric tGMR estimate of 3665 adults (95% CI: 2852 – 4479) with a CV of 11% (Table 3), based on event 1 adult sampling in the lower mainstem river. Using the 306 adults sampled in upriver spawning tributaries in August of 2020 and all 682 sampled parr, COLONY identified 96 unique adult parents assigned to parr offspring and inferred 721 total unique successfully reproducing adults. These quantities produced a hypergeometric tGMR estimate of 2284 adults (95% CI: 1936–2632) with a CV of 8%, based on adult event 2 sampling in the spawning tributaries.

**FIGURE 5:**
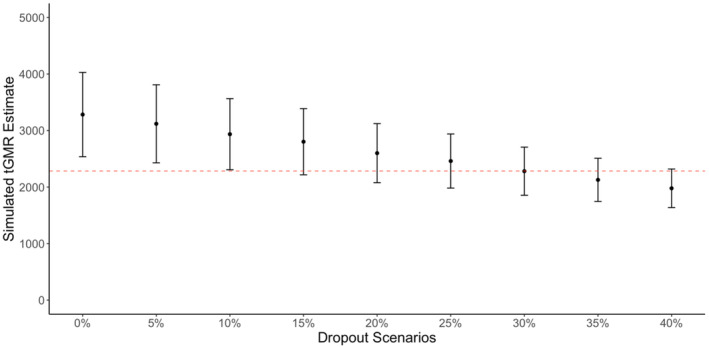
Simulated escapement (*y*‐axis) for Chilkat River Chinook salmon using a single‐sex binomial trans‐generational genetic mark–recapture (tGMR) estimate paramterized with varying rates of dropout (*x*‐axis) between the mainstem and tributary sampling areas from 0% to 40%. Error bars indicate the 95% range of abundances predicted across 1000 iterations for each dropout scenario. We compare these dropout scenarios to our empirical escapement estimate (red dashed line), quantified using a hypergeometric tGMR approach on adults sampled in the upriver spawning tributaries.

**FIGURE 6:**
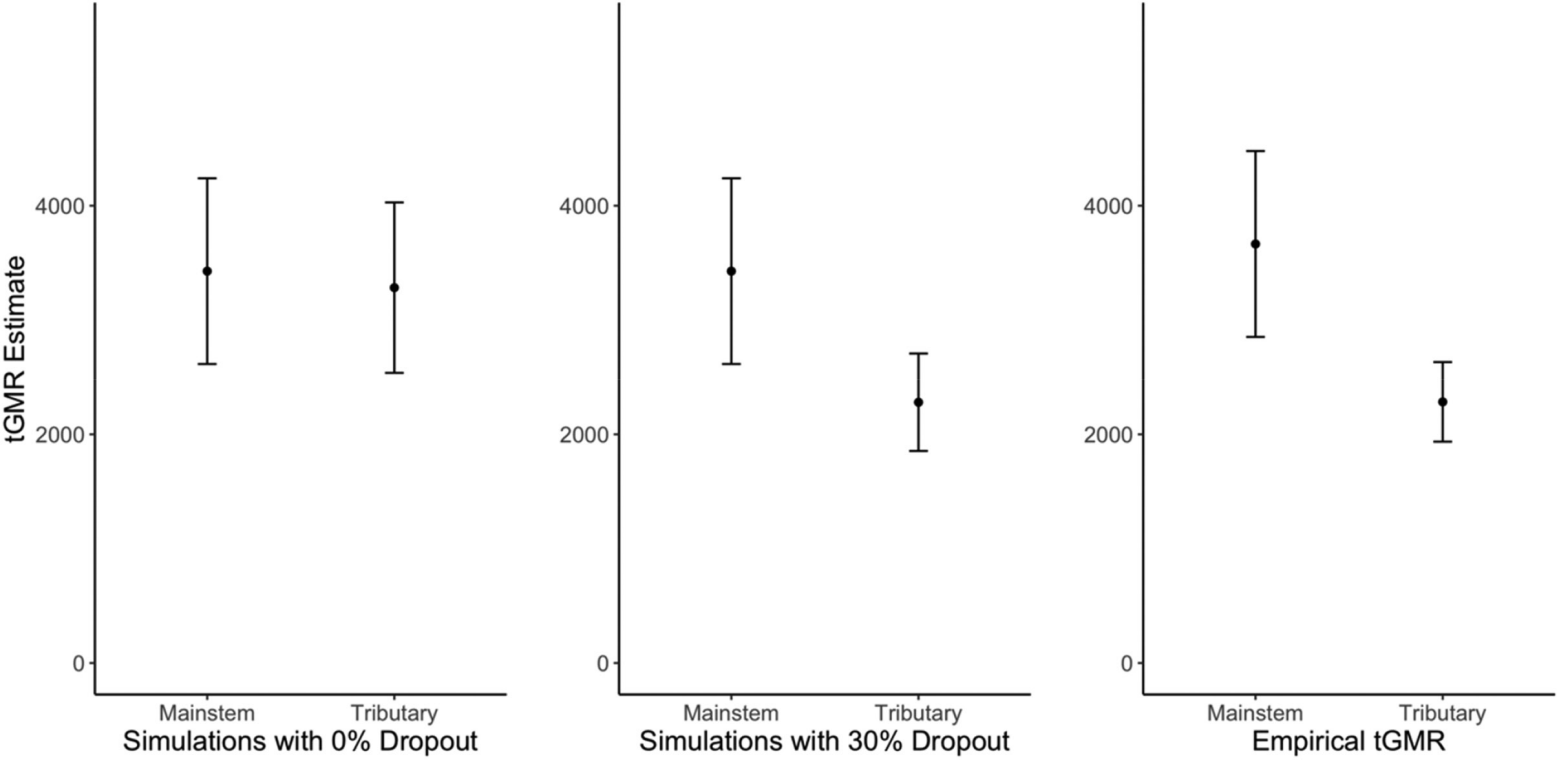
Simulated escapement (*y*‐axes) of Chilkat River Chinook salmon comparing 0% dropout (left) and 30% dropout scenarios (middle) to empirical hypergeometric mainstem and tributary estimates (right), while accounting for differences in age composition and selectivity. Error bars for simulations indicate the 95% range of abundances predicted across 1000 iterations for each scenario; error bars for empirical estimates indicate 95% confidence intervals.

We evaluated how varying levels of dropout between adult sampling locations (lower river mainstem vs upriver tributary spawning grounds) may influence tGMR estimates. As the percentage of adult dropout increased across simulations, the average estimate based on upriver tributary adult sampling, and the range of abundances predicted by 95% of simulations, decreased (Table S5, Figure 5). For example, at 0% dropout, the average abundance estimate was 3283 fish, while at 40% dropout, the average abundance estimate was 1978. Notably, vectors of age‐specific sampling selectivity values (**P**_**sampling**_) used to parameterize the scenarios examining the influence of dropout were calculated from the empirical case study data separately for mainstem **P**_**sampling**_ = [0.18, 0.12, 0.60, and 0.10] and tributary habitats **P**_**sampling**_ = [0.01, 0.34, 0.29, and 0.36]. The simulated differences observed in scenarios in which **Proportion**_**dropout**_ was held at 0% for both lower mainstem river and upriver tributary sampling locations (Figure 6, left panel) are attributable to differences in **P**_**sampling**_. The difference between these estimates was 144 fish (Figure 6, left panel; calculated from Table S5), which was insufficient to explain the empirically observed difference in abundance estimates between the mainstem and tributary habitats (1381 fish, Figure 6 right panel, calculated from Table 3). The simulation scenario with a 30% dropout rate (Figure 6, middle panel) produced an average estimate (2281 fish; 95% predicted range: 1855–2706), which aligned well with the empirical hypergeometric tGMR estimator calculated using only tributary adults (2284 fish; 95% CI: 1936–2632; Table 3 and Table S3). This simulated exploration of varying dropout rates and selectivity differences between adult sampling locations explores one potential mechanism underpinning the differences we observed between our mainstem and tributary empirical tGMR estimates.

## 4 DISCUSSION

We used tGMR to estimate the escapement of Chilkat River Chinook salmon during the 2020 season and developed a simulation framework to evaluate potential biases arising from adult sampling. Our findings suggest tGMR can produce estimates concordant with traditional mark–recapture while providing increased precision, particularly when using adults representatively sampled in the mainstem habitat. Prior tGMR applications have similarly observed increased precision with tGMR estimates in comparison to traditional methods (Rawding et al., 2014; Small et al., 2020; Whitmore, 2016), which arises in part from the high recapture rates afforded by genetically sampling adults via their offspring. A carefully designed, representative adult sampling program is crucial for avoiding violations of key tGMR assumptions (e.g., (1) assuming an equal probability of adult capture and (2) assuming a closed population), and we provide empirical and simulated evidence of bias that can be driven by (1) age‐specific co‐occurring differences in both reproductive success and sampling selectivity and (2) adult dropout between sample sites. Motivated by previous efforts to describe potential biases associated with POP‐based abundance estimates (Waples & Feutry, 2022), we leveraged parameters from our Chilkat River case study to develop an individual‐based model to simulate the expected direction and magnitude of bias that may be encountered in tGMR applications. Through evaluation of our findings, we address key uncertainties in the tGMR framework and highlight circumstances that may benefit from adopting tGMR for salmon abundance estimation.

### 4.1 Estimate evaluation

We compared six tGMR estimates – three hypergeometric and three binomial estimates – to our reference abundance estimate, the traditional mark–recapture project. The hypergeometric estimate using mainstem adults was most concordant with the traditional mark–recapture estimate, yet the tGMR estimate had greater precision. It should be noted that the traditional mark–recapture project estimates the escapement of Chinook salmon ages ≥4 in the Chilkat River, while the tGMR application estimates the escapement of all age‐classes, including jacks (age‐3 males). Jacks were estimated to comprise 18% of the mainstem escapement, suggesting that if the traditional mark–recapture project accounted for jacks, our reference estimate would shift upward by 18%. Subsequently, the mainstem tGMR estimate would then be underestimating the escapement, which may be partially explained by our simulation findings that suggest tGMR will be biased between −10% and −13% when both reproductive success and adult sampling selectivity increase with age. We observed increased reproductive success with age in the Chilkat River, and despite the use of multiple gear types, it is possible that the sampling effort was still biased regarding age, size, and reproductive success.

The binomial tGMR estimate using adults collected in the mainstem showed less agreement with the traditional estimate than the hypergeometric counterpart. This departure can likely be attributed to the binomial estimator being less robust to assumption violations and more prone to biases as a result of following sampling with replacement (Rawding et al., 2014; Small et al., 2020). Considering these observed differences within our case study and results from past tGMR projects, we caution tGMR users from relying on the binomial estimator. Instead, it is preferable to identify an informative panel of genomic markers for the target population to confidently infer unsampled parents during parentage analysis so that the hypergeometric framework may be reliably utilized. The marker panel used here may have led to imperfectly inferred unsampled parents, as evidenced by differences in captures, ‘C’, across our three hypergeometric estimates (705–745 inferred unsampled parents; Table 3). This variation may be driven by the marker panel's incomplete ability to differentiate among pairs of unrelated individuals, half siblings, and full siblings (Figures S3–S5). While simulation results indicated high statistical power of our selected markers to accurately differentiate between pairs of unrelated individuals and full siblings, less power to distinguish between full‐ and half‐siblings may have limited COLONY's ability to accurately reconstruct full‐ and half‐sibling family groups (Whitmore, 2016). The hypergeometric tGMR framework can be strengthened by using marker panels with sufficient power to confidently identify half‐sibling relationships.

The tGMR estimates produced using (1) all adults sampled across the watershed and (2) adults only sampled in tributaries resulted in estimates that were less concordant to the traditional mark‐recapture estimate. These differences emphasize that location, timing, and representativeness of adult sampling is critical when conducting tGMR studies (Peterson et al., 2023). It is known that tributary adult samples were not representative of all Chilkat River spawners, given that the distribution of samples across spawning tributaries varied greatly compared to previous radiotelemetry studies (Ericksen & Chapwell, 2006) and the higher sampling selectivity of larger, older fish. Sampling only a subset of tributary spawning locations violates key assumptions, casting doubt on the veracity of the tributary tGMR estimate.

### 4.2 Unequal probability of adult capture: Violating assumption 1

Covariation in reproductive success and adult sampling selectivity has the potential to violate the core tGMR assumption that all adults have the same probability of being sampled. Results from our simulations indicate that bias from violating this assumption is most severe and variable when both the probability of reproductive success and sampling selectivity differ with age in opposing directions. For example, when reproductive success increases with age and sampling selectivity is biased toward younger individuals (such as when using a fishwheel), then bias can be substantial and positive. However, when both reproductive success and sampling selectivity increase with age simultaneously, simulated bias is only slightly negative. Variation in bias is reduced considerably under these circumstances, as the expected number of POPs encountered when reproductive success and sampling selectivity simultaneously increase with age becomes quite consistent. When these traits vary in opposing directions, the expected number of encountered POPs ranges drastically due to random chance, which has been demonstrated as the primary mechanism driving bias variability in POP‐based estimators (Waples & Feutry, 2022). The largely predictable and minimal nature of the bias we observed when these traits covary increasingly with age makes these circumstances amenable to calculating a tGMR correction factor. Additionally, it is these very conditions (increasing reproductive success and sampling selectivity with age) that are most likely to be encountered when enumerating salmonids. The expectation that older Pacific salmon are more reproductively successful than their younger counterparts is well‐documented (Koch & Narum, 2021) and further supported by our results. Additionally, the most frequently used gear types are biased toward selecting older, more reproductively successful individuals (Elliott & Peterson, 2018). The slight negative bias driven by these co‐occurring conditions will likely not perturb the reliability of tGMR if identified and accounted for, at least within the limited parameter settings presented in this simulation study.

### 4.3 Dropout: Violating assumption 2

An alternative explanation for the differences between the mainstem and tributary tGMR abundance estimates is adult dropout. A study that applied CKMR to a population of iteroparous Atlantic salmon in Norway similarly found that variation in spatial and temporal adult sampling resulted in contrasting estimates of escapement (Wacker et al., 2021). Samples collected near the point of freshwater entry were thought to result in CKMR estimates of total Atlantic salmon escapement, while individuals sampled during spawning surveys were described as producing an estimate of only adults that had successfully migrated to the upriver breeding habitat. The observed parallel differences among these CKMR and tGMR findings highlight that dropout throughout the adult freshwater migration can drive differences in kinship‐based population estimators.

Dropout occurring during freshwater migrations will likely increase as climate change and habitat degradation further reduce optimal conditions for the migration of spawning salmonids (von Biela et al., 2022). While our simulations suggest that a 30% dropout rate is a potential explanation for the discrepancy between empirical mainstem and tributary tGMR estimates, it is important to note that the 95% range of abundances predicted across simulations testing 15%–40% rates of dropout also overlap with the empirical tributary tGMR estimate. A previous radiotelemetry experiment conducted on Chilkat River Chinook salmon in 2005 found a 12% dropout rate (Ericksen & Chapwell, 2006), which is considerably lower than our point estimate for 2020. Previously, the highest recorded dropout rate for Chinook salmon in Southeast Alaska was 23% on the Taku River in 2016 (Richards et al., 2017). Further combining radiotelemetry and tGMR studies may provide a reliable path forward to evaluate change in dropout over time.

### 4.4 Model assumptions and limitations

Our study provides an individual‐based model as a tool to quantify and correct for violations of key tGMR assumptions. However, it should be emphasized that our simulation framework simplifies wild Pacific salmon population dynamics and makes several notable assumptions that may affect the interpretation of findings. First, sex was not considered in our simulation framework; therefore, the model assumes that the focal population has an equal sex ratio and no variation in age‐specific reproductive success or sampling selectivity between sexes. These sex‐based assumptions may result in model outputs that incorrectly estimate tGMR bias and variability. Therefore, future applications of this model should consider whether violations of these assumptions may influence results in study systems with evidence of non‐equal sex ratios and sexual variation in age‐specific reproductive success and sampling selectivity. We chose not to include sex in our model because non‐lethal sex identification of Chilkat River Chinook salmon is error‐prone, and we were uncertain of the validity of the sex metadata associated with our non‐lethal adult samples. Testing the effect of sex ratios and variation in sex‐specific parameters would be a useful extension of our framework and could be tested in a system with reliable sex data to improve the accuracy of future tGMR applications. To increase the feasibility of tGMR, we encourage the continued development of marker panels that include sex‐associated loci for accurate and non‐lethal sex identification among semelparous Pacific salmon (e.g., McKinney et al., 2020).

Our model yields an estimate that is most akin to a single‐sex binomial tGMR estimate, which may limit our ability to compare simulated estimates to empirical hypergeometric estimates. Incorporating the hypergeometric estimator in our simulations would have required explicitly simulating genotypes for each individual, which was beyond the scope of this modeling exercise. Furthermore, the single‐sex binomial estimator is likely reducing the simulated variation in family size compared to populations with discrete sexes. The binomial estimator is dependent on the total number of POPs, which may be underestimated when variation in family size is reduced. Therefore, the single‐sex binomial model may overestimate abundance compared to a wild population with discrete sexes and greater variation in family size. As a result, estimates of dropout calculated in this study may be inflated if fewer POPs are simulated than expected. Future models allowing one to compare binomial and hypergeometric estimators will be useful for resolving these uncertainties.

## 5 CONCLUSIONS

Our empirical case study of Chilkat River Chinook salmon provides support for the use of tGMR as an enumeration tool offering increased precision, accordance with traditional methods, and reduced handling of adult spawners. Choosing between tGMR and traditional mark–recapture methods requires weighing the benefits and costs of each method. Although hardly negligible, the costs of marker development and genotyping continue to decline (Meek & Larson, 2019), and not having to conduct a second adult sampling event is a significant benefit when handling stress is a major concern or when the study system requires expensive and challenging travel to remote locations. A major drawback of tGMR, however, is the delay between adult sampling and the availability of estimates (over a year in Pacific salmon applications to date, including ours). This delay precludes its use for in‐season management and may delay postseason run reconstruction and abundance forecasts for the next season. Ultimately, the trade‐offs between the two methods are likely to be case‐specific, depending on sampling logistics and the information needs for management. Because tGMR has emerged recently, studies that compare both tGMR and traditional methods such as ours and others (Rawding et al., 2014; Small et al., 2020; Whitmore, 2016) are particularly useful for evaluating these trade‐offs.

Our case study and simulation analysis aimed to illuminate uncertainties surrounding violations of (1) the equal probability of capture assumption and (2) the closed population assumption. We assert that these two assumptions are those most likely to be violated when enumerating Pacific salmon; however, further work evaluating the remaining mark–recapture assumptions will continue to inform the utility of tGMR. Specifically, we recommend future efforts investigate the relative importance of random versus non‐random offspring sampling in a tGMR framework, as this may be useful for determining optimal sampling strategies required to achieve unbiased escapement estimates.

The individual‐based model detailed here offers a simple and flexible framework for simulating the accuracy and precision of tGMR estimation across an array of demographic and sampling scenarios. Enumeration of spawning populations using tGMR can provide widely applicable benefits to agencies seeking to increase the effectiveness of escapement‐based management programs, while reducing invasive sampling. Future efforts to enumerate salmonids expressing complex life histories and various population sizes should additionally be explored to determine tGMR's reliability for enumerating a diversity of semelparous populations.

## CONFLICT OF INTEREST STATEMENT

The authors declare no conflict of interest.

## Supporting information


Appendix S1

## Data Availability

Data for this study are available at: https://github.com/swrosenbaum/tGMR_simulations.
