# Supplementary material for: Reliability of trans‐generational genetic mark–recapture (tGMR) for enumerating Pacific salmon
Source: Evol Appl. 2024 Feb 7;17(2):e13647. doi: 10.1111/eva.13647 (PMC10848877; doi:10.1111/eva.13647)
Supplement: Supplementary file 1 — Appendix S1 [file EVA-17-e13647-s001.docx]

# **Supplementary Figures**


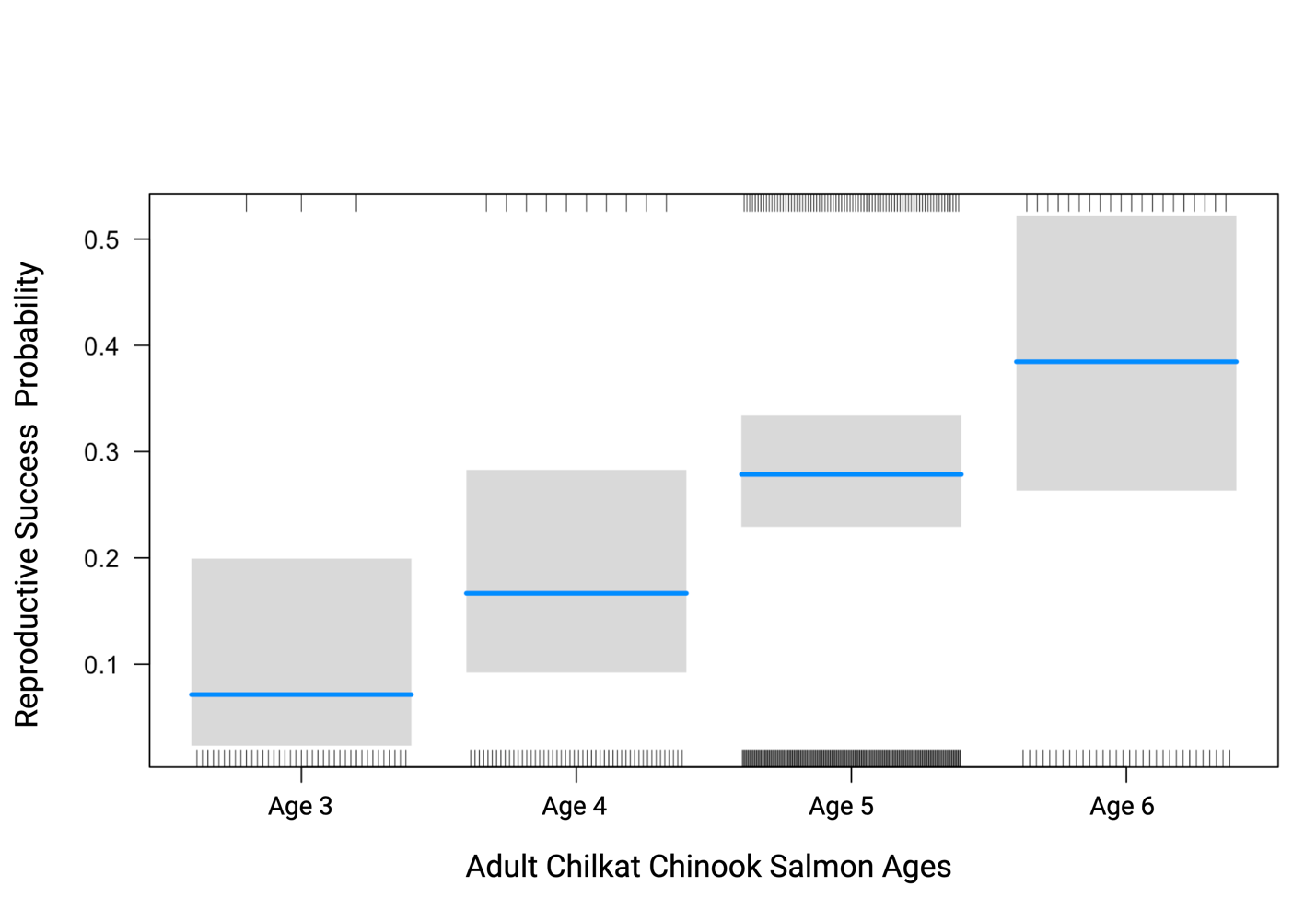


Figure S1: The proportion of individuals that had at least one offspring (y-axis) at a given age (x-axis). Blue horizontal lines indicate the mean **P_RS_** values and the grey shaded area represents the confidence band associated with the mean value.


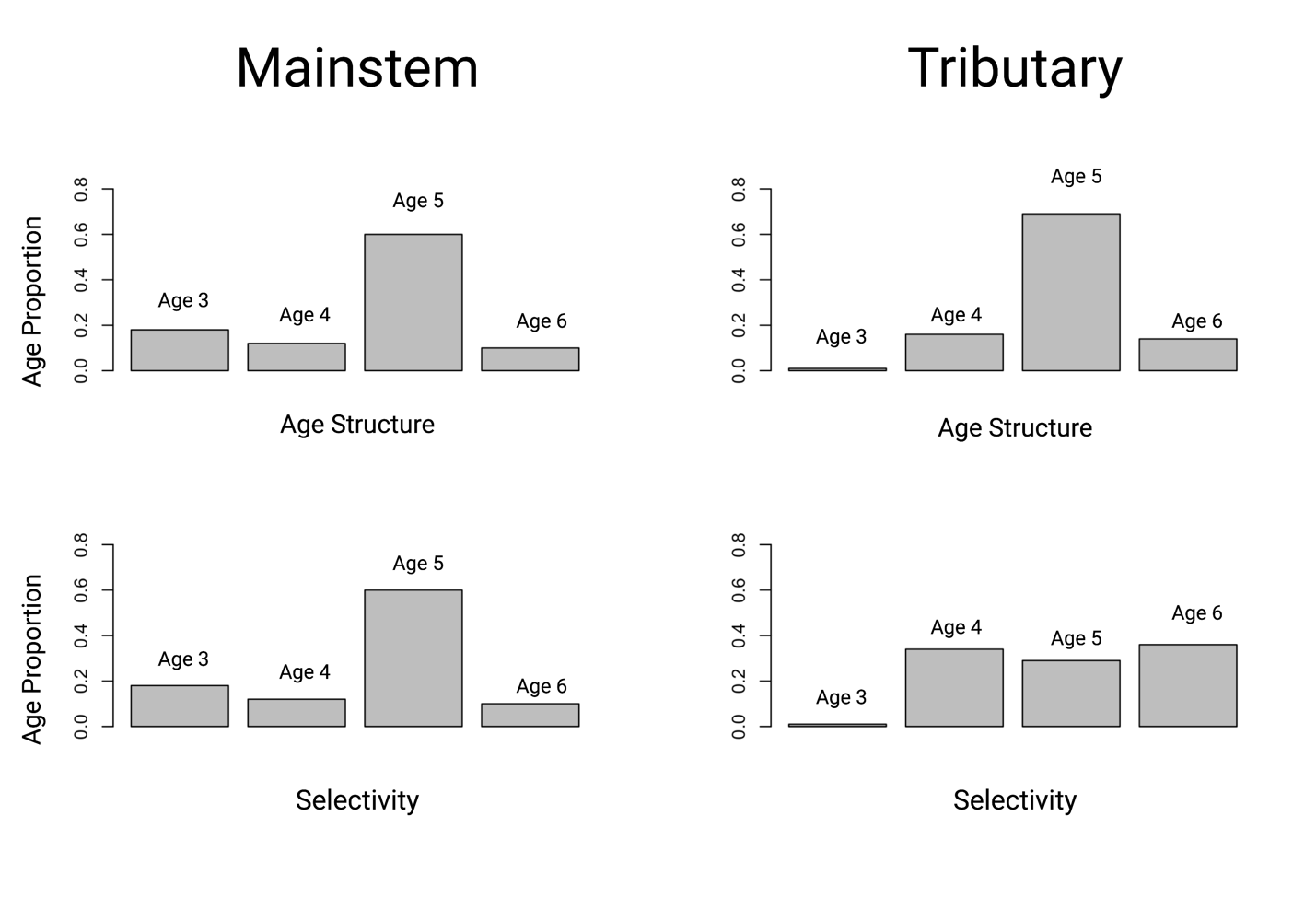


Figure S2: TOP) Proportion of total population size (y-axes) for each age class (x-axes) specific to each sampling habitat (mainstem, left; tributary, right). BOTTOM) Proportion of population sampled (y-axes) for each age class (x-axes) for each sampling habitat (mainstem, left; tributary, right).

**
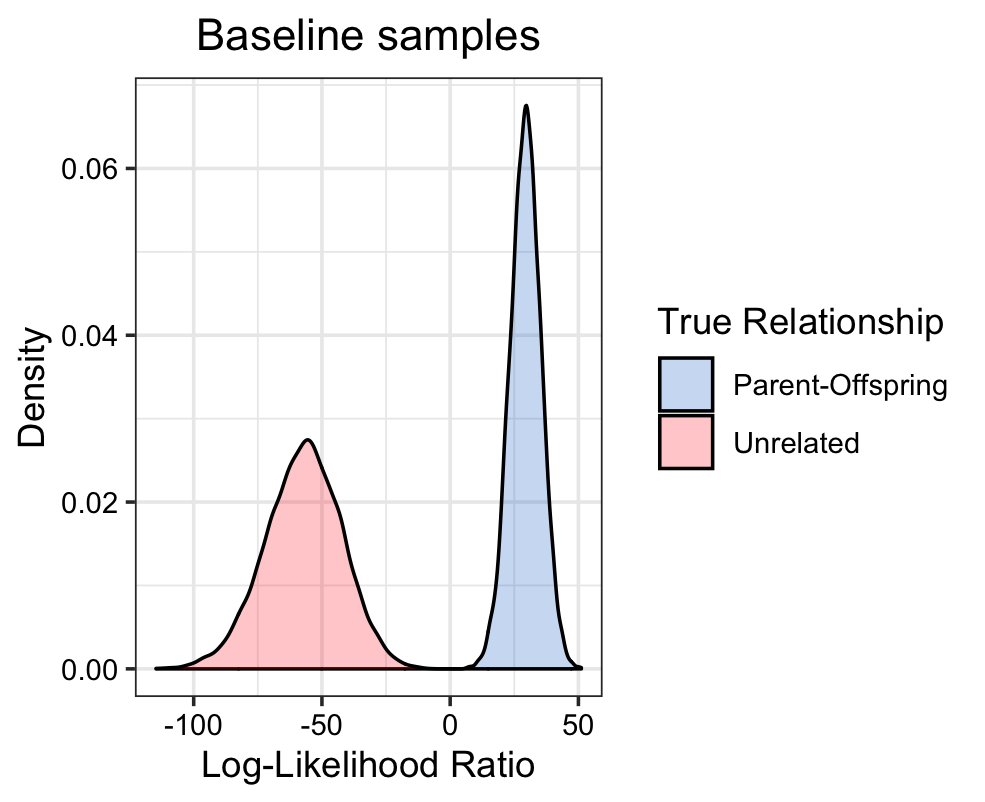
**

Figure S3: Distributions of log-likelihood ratios, generated using CKMRsim, between parent-offspring pairs (blue) and unrelated pairs (red) of Chinook salmon. Baseline samples were genotyped at 301 SNPs and 13 microsatellite loci, however only 255 SNPs were retianed for analyses. Using the available baseline samples from 2004, this CKMRsim anlsysis simulated the power of 255 SNPs plus a single multiplex of 5 microsattelite loci for valid parentage inference.


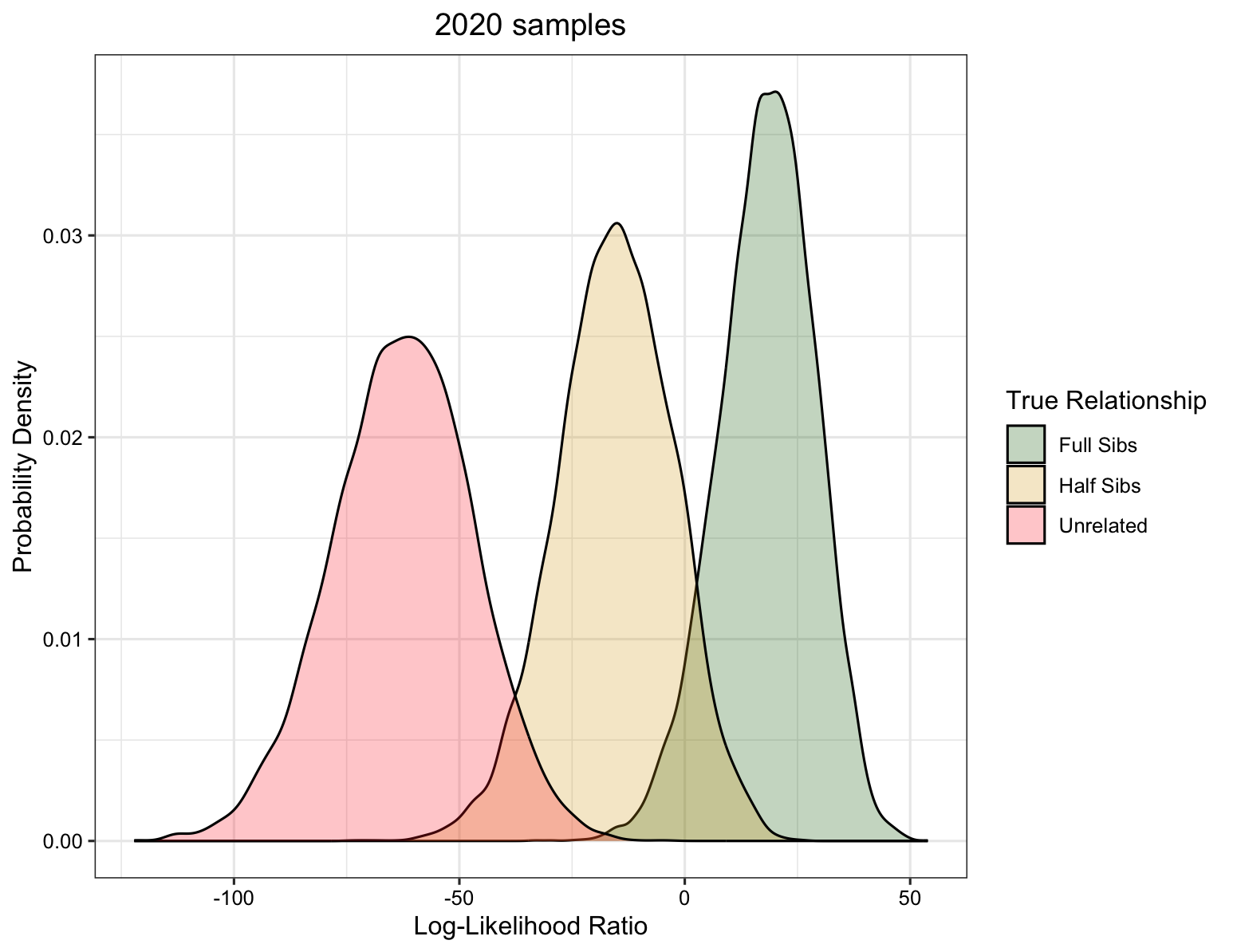


Figure S4: The log-likelihood ratio distribution, generated using CKMRsim, among unrelated pairs (red), half-sibling pairs (yellow), and full-sibling pairs (green) of Chinook salmon. Samples from 2020 were genotyped at 299 SNPs and 5 microsatellite loci. This CKMRsim anlsysis simulated the power of 254 SNPs plus a single multiplex of 5 microsattelite loci for valid sibship inference.


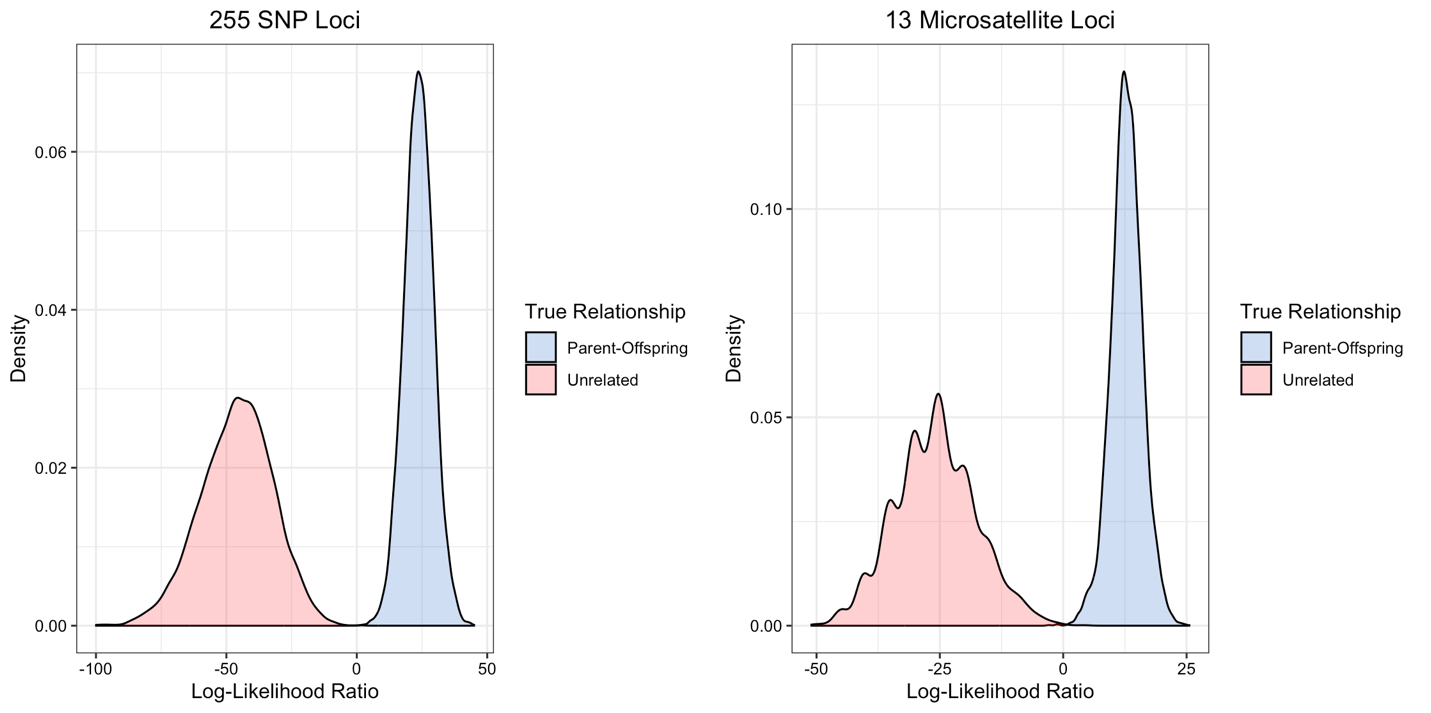


Figure S5: Distributions of log-likelihood ratios, generated using CKMRsim, between parent-offspring pairs (blue) and unrelated pairs (red) of Chinook salmon. The left panel displays log-likelihood distributions for these relationships using 255 SNPs, while the right panel shows the same information using 13 microsatellites.

# **Supplementary Tables**

Table S1: Input parameters for three implmentations of the parentage analysis software, COLONY. The number of Chilkat River Chinook salmon adults when using mainstem samples (implementation 2, not shown) was 295, and the Prob of dam and sire in candidates was 0.09. The number of adults when using tributary samples (implementation 3, not shown) was 306, and the Prob of dam and sire in candidates was 0.10.

| **Parameter** | **Implementation 1: All Adults and All Juveniles** |
| --- | --- |
| Number of adults in the sample | 583 |
| Number of offspring in the sample | 682 |
| Number of loci | 259 |
| Seed for random number generator | 1234 |
| Not updating/updating allele frequency | Updating |
| Dioecious/monoecious species | Dioecious |
| Inbreeding absent/present | Absent |
| Diploid species/haplodiploid species | Diploid |
| Polygamy/monogamy for males & females | Polygamy |
| Clone inference | No |
| Scale full sibship | Yes |
| Sibship prior | Weak |
| Unknown/known population allele frequency | Unknown |
| Number of runs | 1 |
| Length of run | Medium |
| Monitor method | Time in seconds |
| Monitor interval | Seconds |
| Version | Windows |
| Likelihood method | Full-Likelihood |
| Precision | Medium |
| Prob of Dad (and Mum) in candidates | 0.23 |

Table S2: Sample sizes of genoytped Chilkat River Chinook salmon in each sampling event and the number of individuals removed due to missing data, duplicate samples, and excessive heterozygosity. Note that duplicates found in “all adults” are due to recaptures between the mainstem and tributary sampling events.

| **Sample Collection** | **Genotyped** | **Missing Data** | **Duplicates** | **Excessive heterozygosity** | **Final Dataset** |
| --- | --- | --- | --- | --- | --- |
| **All adults** | 641 | 0 | 19 | 39 | 583 |
| **Mainstem Adults** | 312 | 0 | 0 | 17 | 295 |
| **Tributary Adults** | 329 | 0 | 1 | 22 | 306 |
| **Juveniles** | 700 | 10 | 1 | 7 | 682 |

Table S3 Samples sizes and locations for adult Chilkat River Chinook samples collected in 2020 with genotypes passing quality assurance steps.

| Sample Location | Number of Adults in Final Dataset |
| --- | --- |
| Mainstem Fishwheel (Chilkat River) | 150 |
| Mainstem Gillnet (Chilkat River | 145 |
| Kelsall (Chilkat Tributary) | 79 |
| Tahini (Chilkat Tributary) | 210 |
| Klehini (Chilkat Tributary) | 17 |

Table S4 Samples sizes and locations for juvenile Chilkat River Chinook samples collected in 2021 with genotypes passing quality assurance steps.

| Sample Location | Number of Juveniles in Final Dataset |
| --- | --- |
| Mainstem (Chilkat River) | 191 |
| Kelsall (Chilkat Tributary) | 247 |
| Tahini (Chilkat Tributary) | 244 |

Table S5: Key paramters and results for the dropout simulations for Chilkat River Chinook salmon.

| **Scenario** | **P_sampling_** | **Adults** | **Dropout %** | **Estimate** | **95% Range of Abundances** |
| --- | --- | --- | --- | --- | --- |
| 1 (Mainstem) | 0.18, 0.12, 0.60, 0.10 | 295 | 0 | 3,427 | 2,615 – 4,239 |
| 2 (Tributary) | 0.01, 0.34, 0.29, 0.36 | 306 | 0 | 3,283 | 2,538 – 4,028 |
| 3 (Tributary) | 0.01, 0.34, 0.29, 0.36 | 306 | 5 | 3,119 | 2,429 – 3,810 |
| 4 (Tributary) | 0.01, 0.34, 0.29, 0.36 | 306 | 10 | 2,935 | 2,306 – 3,564 |
| 5 (Tributary) | 0.01, 0.34, 0.29, 0.36 | 306 | 15 | 2,802 | 2,216 – 3,388 |
| 6 (Tributary) | 0.01, 0.34, 0.29, 0.36 | 306 | 20 | 2,600 | 2,078 – 3,123 |
| 7 (Tributary) | 0.01, 0.34, 0.29, 0.36 | 306 | 25 | 2,460 | 1,982 – 2,938 |
| 8 (Tributary) | 0.01, 0.34, 0.29, 0.36 | 306 | 30 | 2,281 | 1,855 – 2,706 |
| 9 (Tributary) | 0.01, 0.34, 0.29, 0.36 | 306 | 35 | 2,128 | 1,745 – 2,510 |
| 10 (Tributary) | 0.01, 0.34, 0.29, 0.36 | 306 | 40 | 1,978 | 1,638 – 2,319 |

# **Supplementary Material**

## **Parentage Power Analysis Methods**

We used previously collected baseline genotype data from adult Chilkat River Chinook salmon sampled in 2004 (K. Shedd & Gilk-Baumer, 2021) and the R package *CKMRsim* (Dr. Eric Anderson <https://github.com/eriqande/CKMRsim>) to evaluate the power of various combinations of genetic markers to sufficiently detect all parent-offspring relationships (false negative rate < 0.0001, for a false positive rate = 100 times the reciprocal of the number of anticipated pairwise comparisons of parents and offspring). These available baseline samples were genotyped for 301 single nucleotide polymorphism (SNP) genetic markers using Genotyping-in-Thousands by sequencing (GT-seq; Campbell et al., 2015) in combination with 13 microsatellite loci from the Genetic Analysis of Pacific Salmon (GAPS) panel (Seeb et al., 2007; Moran et al., 2013). The 301 SNP GT-seq panel was based off of the 299 SNP v3.0 GT-seq panel developed by the Columbia River Inter-tribal Fish Commission Hagerman Genetics Laboratory (Hess et al., 2014) with two additional SNPs (*Ots_uwsnp640165* and *Ots_uwsnp670329*) that are associated with the run timing gene GREB1L (Prince et al., 2017). We used *CKMRsim* to calculate population allele frequencies from baseline samples, simulate multi-locus genotypes for different relationships, and evaluate the statistical power of a given marker set (254 SNPs and 5 microsatellite loci) for identifying valid parentage assignments (Figure S3).

## **Parentage Power Analysis Results**

When simulating the false positive and false negative rates of parentage assignments associated with various combinations of GAPS microsatellite multiplexes in conjunction with a GT-seq SNP panel in *CKMRsim*, we determined that a single multiplex of five microsatellite loci accompanied by the SNP panel would provide ample power for valid parentage assignments. Figure 4 shows that the distribution of log-likelihood ratios for POPs versus unrelated pairs have minimal overlap, indicating adequate statistical power for differentiating between these two groups. Our evaluation of parentage panels in *CKMRsim* was based on the criteria of requiring a false positive rate that is 10 times lower than the reciprocal of the number of comparisons we made (0.1 x (641 adults x 700 parr))^-1^ = 2.2^-5^) (recommendation from Eric Anderson, author of *CKMRsim*). Using ADF&G’s baseline samples of Chilkat River Chinook salmon, we observed a simulated false positive rate of 2.34^-7^ and a false negative rate of 0.0006 in *CKMRsim* associated with our proposed panel.

Once the 2020 adult samples were genotyped, we re-ran the *CKMRsim* analysis to evaluate how similar the final panels performed for parentage analysis between the baseline and 2020 samples. As expected, the contemporary *CKMRsim* results indicated that the 2020 samples provided concordant statistical power for parentage inference, simulating a false positive rate of 3.98e-7 and a false negative rate of 0.0001.

# **Data Archiving/Accessibility Statement:** Data for this study are available at: <https://github.com/swrosenbaum/tGMR_simulations>

# **Literature Cited**

Adkison, M. D. (2022). A Review of Salmon Spawner-Recruitment Analysis: The Central Role of the Data and Its Impact on Management Strategy. *Reviews in Fisheries Science & Aquaculture*, *30*(3), 391–427. https://doi.org/10.1080/23308249.2021.1972086

Anderson, J. H., Faulds, P. L., Atlas, W. I., & Quinn, T. P. (2013). Reproductive Success of Captively Bred and Naturally Spawned Chinook Salmon Colonizing Newly Accessible Habitat. *Evolutionary Applications*, *6*(2), 165–179. https://doi.org/10.1111/j.1752-4571.2012.00271.x

Bailey, N. T. J. (1951). On Estimating the Size of Mobile Populations from Recapture Data. *Biometrika*. 293–306.

Beamish, R. (2022). The need to see a bigger picture to understand the ups and downs of Pacific salmon abundances. *ICES Journal of Marine Science, 79,* 1005­–1014­­­. https://doi.org/10.1093/icesjms/fsac036

Berdahl, A. M., Kao, A. B., Flack, A., Westley, P. A. H., Codling, E. A., Couzin, I. D., Dell, A. I., & Biro, D. (2018). Collective animal navigation and migratory culture: From theoretical models to empirical evidence. *Philosophical Transactions of the Royal Society B: Biological Sciences*, *373*(1746), 20170009. https://doi.org/10.1098/rstb.2017.0009

Biela, V. R., Sergeant, C. J., Carey, M. P., Liller, Z., Russell, C., Quinn‐Davidson, S., Rand, P. S., Westley, P. A. H., & Zimmerman, C. E. (2022). Premature Mortality Observations among Alaska’s Pacific Salmon During Record Heat and Drought in 2019. *Fisheries*, *47*(4), 157–168. https://doi.org/10.1002/fsh.10705

Bonner, S. J., Morgan, B. J. T., & King, R. (2010). Continuous Covariates in Mark-Recapture-Recovery Analysis: A Comparison of Methods. *Biometrics*, *66*(4), 1256–1265. https://doi.org/10.1111/j.1541-0420.2010.01390.x

Bowerman, T., Keefer, M. L., & Caudill, C. C. (2016). Pacific Salmon Prespawn Mortality: Patterns, Methods, and Study Design Considerations. *Fisheries*, *41*(12), 738–749. https://doi.org/10.1080/03632415.2016.1245993

Bravington, M. V., Skaug, H. J., & Anderson, E. C. (2016). Close-Kin Mark-Recapture. *Statistical Science*, *31*(2). https://doi.org/10.1214/16-STS552

Bromaghin, J. F. (2005). A Versatile Net Selectivity Model, With Application to Pacific Salmon and Freshwater Species of the Yukon River, Alaska. *Fisheries Research*, *74*(1–3), 157–168. https://doi.org/10.1016/j.fishres.2005.03.004

Campbell, N. R., Harmon, S. A., & Narum, S. R. (2015). Genotyping‐in‐Thousands by Sequencing (GT‐seq): A Cost Effective SNP Genotyping Method Based on Custom Amplicon Sequencing. *Molecular Ecology Resources*, *15*(4), 855–867. https://doi.org/10.1111/1755-0998.12357

Chapman, D. G. (1951). Some Properties of Hypergeometric Distribution with Application to Zoological Census. *University of California Publications Statistics*, *1*, 131–160.

Chapell, R. S. 2014. Production, escapement, and juvenile tagging of Chilkat River Chinook salmon in 2011. Alaska Department of Fish and Game, Fishery Data Series No. 14-55, Anchorage.

Chinook Technical Committee. (2023). Annual Report of Catch and Escapement. For 2022. Pacific Salmon Commission. Report TCCHINOOK, (23)-02. Vancouver, BC.

Clark, J. H., McGregor, A., Mecum, R. D., Krasnowski, P., & Carroll, A. M. (2006). *The Commercial Salmon Fishery in Alaska*. *Alaska Fishery Research Bulletin,* 12(1), 1–46.

Elliott, B. W. (2022). *Operational plan: Chilkat River Chinook Salmon Escapement Studies in 2020.* 68. Alaska Department of Fish and Game, Division of Sport Fish, Regional Operational Plan No. ROP.SF.1J.2022.06, Anchorage.

Elliott, B. W., & Peterson, R. L. (2018). *Production and Harvest of Chilkat River Chinook and Coho Salmon,* 2018–2019. Alaska Department of Fish and Game, Regional Operational Plan No. ROP.SF.1J.2018.10, Anchorage.

Elliott, B. W., & Peterson, R. L. (2022). *Production and Harvest of Chilkat River Chinook and Coho Salmon, 2020*–2021. Alaska Department of Fish and Game, Division of Sport Fish, Regional Operational Plan No. ROP.SF.1J.2022.26, Anchorage.

Ericksen, R. P., and McPherson, S. A. (2004). *Optimal Production of Chinook Salmon from the Chilkat River*. Alaska Department of Fish and Game, Fishery Manuscript No. 04-01, Anchorage.

Ericksen, R. P., and Chapwell, R. S. (2006). *Production and spawning distribution of Chilkat. River Chinook salmon in 2005*. Alaska Department of Fish and Game, Fishery Data Series No. 06–76, Anchorage.

Helfield, J. M., & Naiman, R. J. (2006). Keystone Interactions: Salmon and Bear in Riparian Forests of Alaska. *Ecosystems*, *9*(2), 167–180. https://doi.org/10.1007/s10021-004-0063-5

Hess, J. E., Campbell, N. R., Matala, A. P., & Narum, S. R. (2014). *Genetic Assessment of Columbia River Stocks*. Columbia River Inter-Tribal Fish Commission annual report.

Koch, I. J., & Narum, S. R. (2021). An Evaluation of the Potential Factors Affecting Lifetime Reproductive Success in Salmonids. *Evolutionary Applications*, *14*(8), 1929–1957. https://doi.org/10.1111/eva.13263

Koo, T. S. Y. (1955). Biology of the red salmon, *Oncorhynchus nerka* (Walbaum), of Bristol Bay, Alaska, as revealed by a study of their scales. Ph.D. thesis, University of Washington, Seattle.

Lin, J. E., Hard, J. J., Hilborn, R., & Hauser, L. (2017). Modeling local adaptation and gene flow in sockeye salmon. *Ecosphere*, *8*(12). https://doi.org/10.1002/ecs2.2039

Lum, J. L., and Fair. L. (2018). *Chilkat River and King Salmon River King Salmon Stock Status and Action Plan, 2018*. Alaska Department of Fish and Game, Regional Information Report No 1J18-05, Douglas

May, S. A., Hard, J. J., Ford, M. J., Naish, K. A., & Ward, E. J. (2023). Assortative Mating for Reproductive Timing Affects Population Recruitment and Resilience in a Quantitative Genetic Model. *Evolutionary Applications*, *16*(3), 657–672. https://doi.org/10.1111/eva.13524

McKinney, G. J., Pascal, C. E., Templin, W. D., Gilk-Baumer, S. E., Dann, T. H., Seeb, L. W., & Seeb, J. E. (2020). Dense SNP Panels Resolve Closely Related Chinook Salmon Populations. *Canadian Journal of Fisheries and Aquatic Sciences*, *77*(3), 451–461. https://doi.org/10.1139/cjfas-2019-0067

McPherson, S., Bernard, D., Clark, J. H., Pahlke, K., Jones, E., Hovanisian, J. D., Weller, J., &. Ericksen, R. (2003). *Stock Status and Escapement Goals for Chinook Salmon Stocks in Southeast Alaska*. Alaska Department of Fish and Game, Special Publication No. 03-01, Anchorage.

Meek, M. H., & Larson, W. A. (2019). The Future is Now: Amplicon Sequencing and Sequence Capture Usher in the Conservation Genomics Era. *Molecular Ecology Resources*, *19*(4), 795–803. https://doi.org/10.1111/1755-0998.12998

Moore, J. W. (2006). Animal Ecosystem Engineers in Streams. *BioScience*, *56*(3), 237–246.

Moran, P., Teel, D. J., Banks, M. A., Beacham, T. D., Bellinger, M. R., Blankenship, S. M., Candy, J. R., Garza, J. C., Hess, J. E., Narum, S. R., Seeb, L. W., Templin, W. D., Wallace, C. G., & Smith, C. T. (2013). Divergent Life-history Races Do Not Represent Chinook Salmon Coast-Wide: The importance of Scale in Quaternary Biogeography. *Canadian Journal of Fisheries and Aquatic Sciences*, *70*(3), 415–435. https://doi.org/10.1139/cjfas-2012-0135

Naiman, R. J., Bilby, R. E., Schindler, D. E., & Helfield, J. M. (2002). Pacific Salmon, Nutrients, and the Dynamics of Freshwater and Riparian Ecosystems. *Ecosystems*, *5*(4), 399–417. https://doi.org/10.1007/s10021-001-0083-3

Parsons, A., & Skalski, J. (2010). Quantitative Assessment of Salmonid Escapement Techniques. *Reviews in Fisheries Sciences*, *18.4*, 301–314.

Peterson, R., Shedd, K., Frost, N., Elliott, B., & Richards, P. (2023). *Estimating Adult Chinook Salmon Abundance on the Chilkat and Unuk Rivers Using Transgenerational Genetic Mark–Recapture*. Alaska Department of Fish and Game, Divisions of Sport Fish and Commercial Fisheries, Regional Operational Plan No. ROP.SF.1J.2023.02, Douglas.

Piccolo, J. J., Adkison, M. D., & Rue, F. (2009). Linking Alaskan Salmon Fisheries Management with Ecosystem-based Escapement Goals: A Review and Prospectus. *Fisheries*, *34*(3), 124–134. https://doi.org/10.1577/1548-8446-34.3.124

Pradel, R. (1996). Utilization of Capture-Mark-Recapture for the Study of Recruitment and Population Growth Rate. *Biometrics*, *52*(2), 703. https://doi.org/10.2307/2532908

Prince, D. J., Saglam, I. K., Hotaling, T. J., Spidle, A. P., & Miller, M. R. (2017). The Evolutionary Basis of Premature Migration in Pacific Salmon Highlights the Utility of Genomics for Informing Conservation. Science Advances, 3(8), e1603198.

R Core Team (2023). _R: A Language and Environment for Statistical Computing_. R

Foundation for Statistical Computing, Vienna, Austria. <https://www.R-project.org/>.

Rawding, D. J., Sharpe, C. S., & Blankenship, S. M. (2014). Genetic-Based Estimates of Adult Chinook Salmon Spawner Abundance from Carcass Surveys and Juvenile Out-Migrant Traps. *Transactions of the American Fisheries Society*, *143*(1), 55–67. https://doi.org/10.1080/00028487.2013.829122

Reed, T. E., Schindler, D. E., Hague, M. J., Patterson, D. A., Meir, E., Waples, R. S., & Hinch, S. G. (2011). Time to Evolve? Potential Evolutionary Responses of Fraser River Sockeye Salmon to Climate Change and Effects on Persistence. *PLoS ONE*, *6*(6), e20380. https://doi.org/10.1371/journal.pone.0020380

Reynolds, J. H., Woody, C. A., Gove, N. E., & Fair, L. F. (2007). Efficiently Estimating Salmon Escapement Uncertainty Using Systematically Sampled Data. *American Fisheries Society*, *54*.

Richards, P., Williams, J., Power, S. J. H., Boyce, I., & Foos, A. (2017). Migration, Tagging Response, and Distribution of Chinook Salmon Returning to the Taku River, 2018*. Alaska Department of Fish and Game, Division of Sport Fish, Regional Operational Plan ROP.SF.1J.2018.06, Anchorage.*

Riddell, B.E., Howard, K.G., Munro, A.R. (2022). Salmon returns in the Northeast Pacific in relation to expedition observations (and next steps)*. North Pacific Anadromous Fish Commission Technical Report 115-139.*

Roff, D. A. (1973). On the Accuracy of Some Mark-Recapture Estimators. *Oecologia*, *12*(1), 15–34. https://doi.org/10.1007/BF00345468

Rousset, F. (2008). genepop’007: a complete re-implementation of the genepop software for Windows and Linux. *Molecular Ecology Resources*, 8, 103-106. https://doi.org/10.1111/j.1471-8286.2007.01931.x

Sard, N. M., O’Malley, K. G., Jacobson, D. P., Hogansen, M. J., Johnson, M. A., & Banks, M. A. (2015). Factors Influencing Spawner Success in a Spring Chinook Salmon (*Oncorhynchus tshawytscha*) Reintroduction Program. *Canadian Journal of Fisheries and Aquatic Sciences*, *72*(9), 1390–1397. https://doi.org/10.1139/cjfas-2015-0007

Seber, G. A. F. (1982). *The Estimation of Animal Abundance and Related Parameters*.

Seber, G. A. F., & Felton, R. (1981). *Tag Loss and the Petersen Mark-Recapture Experiment*. 10.

Seeb, L., Antonovich, A., Banks, M., Beacham, T., Bellinger, M., Blankenship, S., Campbell, M., Decovich, N., Garza, J., Guthrie, C., & Lundrigan, T. (2007). Development of Standardized DNA Database for Chinook Salmon. *Fisheries*.

Sethi, S. A., & Tanner, T. (2014). Spawning distribution and abundance of a northern Chinook salmon population. *Fisheries Management and Ecology,* 21(6), 427­­–438. https://doi.org/10.1111/fme.12091

Shedd, K., & Gilk-Baumer, S. (2021). *Chinook Salmon Genetic Baseline Update for Southeast Alaska and Canadian AABM fisheries* (Final Report NF-2019-I-10A; PSC Northern Fund). Alaska Department of Fish and Game - Gene Conservation Laboratory.

Shedd, K. R., Lescak, E. A., Habicht, C., Knudsen, E. E., Dann, T. H., Hoyt, H. A., Prince, D. J., & Templin, W. D. (2022). Reduced Relative Fitness in Hatchery‐Origin Pink Salmon in Two Streams in Prince William Sound, Alaska. *Evolutionary Applications*, *15*(3), 429–446. https://doi.org/10.1111/eva.13356

Small, M. P., Scofield, C., Griffith, J., Spidle, A., Verhey, P., & Bowman, C. (2020). *2018 Broodyear Report: Abundance estimates for Stillaguamish River Chinook Salmon Using Trans-Generational Genetic Mark Recapture*. 44.

Wacker, S., Skaug, H. J., Forseth, T., Solem, Ø., Ulvan, E. M., Fiske, P., & Karlsson, S. (2021). Considering Sampling Bias in Close‐Kin Mark–Recapture Abundance Estimates of Atlantic Salmon. *Ecology and Evolution*, *11*(9), 3917–3932. https://doi.org/10.1002/ece3.7279

Wang, J., & Santure, A. W. (2009). Parentage and Sibship Inference From Multilocus Genotype Data Under Polygamy. *Genetics*, *181*(4), 1579–1594. https://doi.org/10.1534/genetics.108.100214

Waples, R. S. (2022).  *TheWeight* : A Simple and Flexible Algorithm for Simulating Non‐Ideal, Age‐Structured Populations. *Methods in Ecology and Evolution*, *13*(9), 2030–2041. https://doi.org/10.1111/2041-210X.13926

Waples, R. S., & Feutry, P. (2022). Close‐Kin Methods to Estimate Census Size and Effective Population Size. *Fish and Fisheries*, *23*(2), 273–293. https://doi.org/10.1111/faf.12615

Whitmore, R. W. (2016). Evaluation of Parameter Estimation and Field Application of Transgenerational Genetic Mark-Recapture. Humboldt State University.

Wingfield, J. C., & Sapolsky, R. M. (2003). Reproduction and Resistance to Stress: When and How. *Journal of Neuroendocrinology*, *15*(8), 711–724. https://doi.org/10.1046/j.1365-2826.2003.01033.x

Yeakel, J. D., Gibert, J. P., Gross, T., Westley, P. A. H., & Moore, J. W. (2018). Eco-evolutionary dynamics, density-dependent dispersal and collective behaviour: Implications for salmon metapopulation robustness. *Philosophical Transactions of the Royal Society B: Biological Sciences*, *373*(1746), 20170018. https://doi.org/10.1098/rstb.2017.0018

Zhou, S. (2002). Size‐Dependent Recovery of Chinook Salmon in Carcass Surveys. *Transactions of the American Fisheries Society*, 9.

# **Appendix**

Appendix S1: Single nucleotide polymorphism and microsatellite loci used to genotype Chilkat River Chinook salmon.

| Locus | Source |
| --- | --- |
| ARNT | Hess et al., 2014 |
| GTH2B-550 | Hess et al., 2014 |
| NOD1 | Hess et al., 2014 |
| Ots_100884-287 | Hess et al., 2014 |
| Ots_101119-381 | Hess et al., 2014 |
| Ots_101554-407 | Hess et al., 2014 |
| Ots_101704-143 | Hess et al., 2014 |
| Ots_101770-82 | Hess et al., 2014 |
| Ots_102213-210 | Hess et al., 2014 |
| Ots_102414-395 | Hess et al., 2014 |
| Ots_102457-132 | Hess et al., 2014 |
| Ots_102801-308 | Hess et al., 2014 |
| Ots_102867-609 | Hess et al., 2014 |
| Ots_103041-52 | Hess et al., 2014 |
| Ots_103122-180 | Hess et al., 2014 |
| Ots_104048-194 | Hess et al., 2014 |
| Ots_104415-88 | Hess et al., 2014 |
| Ots_105105-613 | Hess et al., 2014 |
| Ots_105132-200 | Hess et al., 2014 |
| Ots_105385-421 | Hess et al., 2014 |
| Ots_105401-325 | Hess et al., 2014 |
| Ots_105407-117 | Hess et al., 2014 |
| Ots_105897-124 | Hess et al., 2014 |
| Ots_106313-729 | Hess et al., 2014 |
| Ots_106499-70 | Hess et al., 2014 |
| Ots_106747-239 | Hess et al., 2014 |
| Ots_107074-284 | Hess et al., 2014 |
| Ots_107285-93 | Hess et al., 2014 |
| Ots_107607-315 | Hess et al., 2014 |
| Ots_107806-821 | Hess et al., 2014 |
| Ots_108007-208 | Hess et al., 2014 |
| Ots_108390-329 | Hess et al., 2014 |
| Ots_108735-302 | Hess et al., 2014 |
| Ots_108820-336 | Hess et al., 2014 |
| Ots_109525-816 | Hess et al., 2014 |
| Ots_109693-392 | Hess et al., 2014 |
| Ots_110064-383 | Hess et al., 2014 |
| Ots_110201-363 | Hess et al., 2014 |
| Ots_110381-164 | Hess et al., 2014 |
| Ots_110551-64 | Hess et al., 2014 |
| Ots_110689-218 | Hess et al., 2014 |
| Ots_111084b-619 | Hess et al., 2014 |
| Ots_111312-435 | Hess et al., 2014 |
| Ots_111681-657 | Hess et al., 2014 |
| Ots_112208-722 | Hess et al., 2014 |
| Ots_112301-43 | Hess et al., 2014 |
| Ots_112419-131 | Hess et al., 2014 |
| Ots_112876-371 | Hess et al., 2014 |
| Ots_113242-216 | Hess et al., 2014 |
| Ots_113457-40R | Hess et al., 2014 |
| Ots_115987-325 | Hess et al., 2014 |
| Ots_117242-136 | Hess et al., 2014 |
| Ots_117259-271 | Hess et al., 2014 |
| Ots_117370-471 | Hess et al., 2014 |
| Ots_117432-409 | Hess et al., 2014 |
| Ots_118175-479 | Hess et al., 2014 |
| Ots_118205-61 | Hess et al., 2014 |
| Ots_118938-325 | Hess et al., 2014 |
| Ots_120950-417 | Hess et al., 2014 |
| Ots_122414-56 | Hess et al., 2014 |
| Ots_123048-521 | Hess et al., 2014 |
| Ots_123921-111 | Hess et al., 2014 |
| Ots_124774-477 | Hess et al., 2014 |
| Ots_127236-62 | Hess et al., 2014 |
| Ots_127760-569 | Hess et al., 2014 |
| Ots_128302-57 | Hess et al., 2014 |
| Ots_128693-461 | Hess et al., 2014 |
| Ots_128757-61R | Hess et al., 2014 |
| Ots_129144-472 | Hess et al., 2014 |
| Ots_129170-683 | Hess et al., 2014 |
| Ots_129458-451 | Hess et al., 2014 |
| Ots_129870-55 | Hess et al., 2014 |
| Ots_130720-99 | Hess et al., 2014 |
| Ots_131460-584 | Hess et al., 2014 |
| Ots_131802-393 | Hess et al., 2014 |
| Ots_131906-141 | Hess et al., 2014 |
| Ots_2KER-137 | Hess et al., 2014 |
| Ots_94857-232R | Hess et al., 2014 |
| Ots_94903-99R | Hess et al., 2014 |
| Ots_96222-525 | Hess et al., 2014 |
| Ots_96500-180 | Hess et al., 2014 |
| Ots_96899-357R | Hess et al., 2014 |
| Ots_97077-179R | Hess et al., 2014 |
| Ots_97660-56 | Hess et al., 2014 |
| Ots_98409-850 | Hess et al., 2014 |
| Ots_98683-796 | Hess et al., 2014 |
| Ots_99550-204 | Hess et al., 2014 |
| Ots_aldb-177M | Hess et al., 2014 |
| Ots_AldB1-122 | Hess et al., 2014 |
| Ots_AldoB4-183 | Hess et al., 2014 |
| Ots_arp-436 | Hess et al., 2014 |
| Ots_AsnRS-72 | Hess et al., 2014 |
| Ots_aspat-196 | Hess et al., 2014 |
| Ots_CCR7 | Hess et al., 2014 |
| Ots_CD59-2 | Hess et al., 2014 |
| Ots_CD63 | Hess et al., 2014 |
| Ots_cgo24-22 | Hess et al., 2014 |
| Ots_Chin30up-211 | Hess et al., 2014 |
| Ots_CirpA | Hess et al., 2014 |
| Ots_cox1-241 | Hess et al., 2014 |
| Ots_CRB211 | Hess et al., 2014 |
| Ots_crRAD10447-25 | Hess et al., 2014 |
| Ots_crRAD11620-55 | Hess et al., 2014 |
| Ots_crRAD12037-39 | Hess et al., 2014 |
| Ots_crRAD12711-37 | Hess et al., 2014 |
| Ots_crRAD13725-51 | Hess et al., 2014 |
| Ots_crRAD16540-50 | Hess et al., 2014 |
| Ots_crRAD17527-58 | Hess et al., 2014 |
| Ots_crRAD18289-33 | Hess et al., 2014 |
| Ots_crRAD18492-65 | Hess et al., 2014 |
| Ots_crRAD18937-60 | Hess et al., 2014 |
| Ots_crRAD20262-46 | Hess et al., 2014 |
| Ots_crRAD20376-66 | Hess et al., 2014 |
| Ots_crRAD20887-70 | Hess et al., 2014 |
| Ots_crRAD21115-24 | Hess et al., 2014 |
| Ots_crRAD22960-32 | Hess et al., 2014 |
| Ots_crRAD23631-48 | Hess et al., 2014 |
| Ots_crRAD25367-50 | Hess et al., 2014 |
| Ots_crRAD255-59 | Hess et al., 2014 |
| Ots_crRAD26081-28 | Hess et al., 2014 |
| Ots_crRAD26165-69 | Hess et al., 2014 |
| Ots_crRAD27164-55 | Hess et al., 2014 |
| Ots_crRAD27515-69 | Hess et al., 2014 |
| Ots_crRAD2806-42 | Hess et al., 2014 |
| Ots_crRAD28677-65 | Hess et al., 2014 |
| Ots_crRAD292-21 | Hess et al., 2014 |
| Ots_crRAD33054-62 | Hess et al., 2014 |
| Ots_crRAD33491-71 | Hess et al., 2014 |
| Ots_crRAD34397-33 | Hess et al., 2014 |
| Ots_crRAD35313-66 | Hess et al., 2014 |
| Ots_crRAD36072-29 | Hess et al., 2014 |
| Ots_crRAD36152-44 | Hess et al., 2014 |
| Ots_crRAD38095-29 | Hess et al., 2014 |
| Ots_crRAD38746-36 | Hess et al., 2014 |
| Ots_crRAD42058-48 | Hess et al., 2014 |
| Ots_crRAD44588-67 | Hess et al., 2014 |
| Ots_crRAD46081-56 | Hess et al., 2014 |
| Ots_crRAD46751-42 | Hess et al., 2014 |
| Ots_crRAD47297-55 | Hess et al., 2014 |
| Ots_crRAD5061-27 | Hess et al., 2014 |
| Ots_crRAD55400-59 | Hess et al., 2014 |
| Ots_crRAD55475-26 | Hess et al., 2014 |
| Ots_crRAD57376-68 | Hess et al., 2014 |
| Ots_crRAD57520-66 | Hess et al., 2014 |
| Ots_crRAD57687-34 | Hess et al., 2014 |
| Ots_crRAD60614-46 | Hess et al., 2014 |
| Ots_crRAD60620-51 | Hess et al., 2014 |
| Ots_crRAD61523-71 | Hess et al., 2014 |
| Ots_crRAD66330-60 | Hess et al., 2014 |
| Ots_crRAD69327-53 | Hess et al., 2014 |
| Ots_crRAD73823-60 | Hess et al., 2014 |
| Ots_crRAD74766-28 | Hess et al., 2014 |
| Ots_crRAD75581-70 | Hess et al., 2014 |
| Ots_crRAD78968-46 | Hess et al., 2014 |
| Ots_crRAD92420-25 | Hess et al., 2014 |
| Ots_crRAD9615-69 | Hess et al., 2014 |
| Ots_DDX5-171 | Hess et al., 2014 |
| Ots_EndoRB1-486 | Hess et al., 2014 |
| Ots_EP-529 | Hess et al., 2014 |
| Ots_Est1363 | Hess et al., 2014 |
| Ots_Est740 | Hess et al., 2014 |
| Ots_ETIF1A | Hess et al., 2014 |
| Ots_FARSLA-220 | Hess et al., 2014 |
| Ots_FGF6A | Hess et al., 2014 |
| Ots_FGF6B | Hess et al., 2014 |
| Ots_GCSH | Hess et al., 2014 |
| Ots_GDH-81x | Hess et al., 2014 |
| Ots_GH2 | Hess et al., 2014 |
| Ots_GNRH2-278 | Hess et al., 2014 |
| Ots_GPDH | Hess et al., 2014 |
| Ots_GPH-318 | Hess et al., 2014 |
| Ots_GST-207 | Hess et al., 2014 |
| Ots_GST-375 | Hess et al., 2014 |
| Ots_HFABP-34 | Hess et al., 2014 |
| Ots_HMGB1-73 | Hess et al., 2014 |
| Ots_hnRNPL-533 | Hess et al., 2014 |
| Ots_hsc71-3prime-488 | Hess et al., 2014 |
| Ots_hsc71-5prime-453 | Hess et al., 2014 |
| Ots_hsp27b-150 | Hess et al., 2014 |
| Ots_Hsp90a | Hess et al., 2014 |
| Ots_HSP90B-100 | Hess et al., 2014 |
| Ots_IGF1-91 | Hess et al., 2014 |
| Ots_IK1-328 | Hess et al., 2014 |
| Ots_IL11 | Hess et al., 2014 |
| Ots_IsoT | Hess et al., 2014 |
| Ots_LEI-292 | Hess et al., 2014 |
| Ots_mapK-3prime-309 | Hess et al., 2014 |
| Ots_mapKpr-151 | Hess et al., 2014 |
| Ots_MetA | Hess et al., 2014 |
| Ots_mybp-85 | Hess et al., 2014 |
| Ots_Myc-366 | Hess et al., 2014 |
| Ots_myo1a-384 | Hess et al., 2014 |
| Ots_myoD-364 | Hess et al., 2014 |
| Ots_nelfd-163 | Hess et al., 2014 |
| Ots_NFYB-147 | Hess et al., 2014 |
| Ots_nkef-192 | Hess et al., 2014 |
| Ots_nramp-321 | Hess et al., 2014 |
| Ots_ntl-255 | Hess et al., 2014 |
| Ots_OPLW-173 | Hess et al., 2014 |
| Ots_OPSW-152 | Hess et al., 2014 |
| Ots_Ostm1 | Hess et al., 2014 |
| Ots_OTALDBINT1-SNP1 | Hess et al., 2014 |
| Ots_OTNAML12_1-SNP1 | Hess et al., 2014 |
| Ots_Ots311-101x | Hess et al., 2014 |
| Ots_OTSBMP-2-SNP1 | Hess et al., 2014 |
| Ots_OTSMTA-SNP1 | Hess et al., 2014 |
| Ots_OTSTF1-SNP1 | Hess et al., 2014 |
| Ots_P450 | Hess et al., 2014 |
| Ots_P450-288 | Hess et al., 2014 |
| Ots_P53 | Hess et al., 2014 |
| Ots_parp3-286 | Hess et al., 2014 |
| Ots_PEMT | Hess et al., 2014 |
| Ots_pigh-105 | Hess et al., 2014 |
| Ots_pop5-96 | Hess et al., 2014 |
| Ots_ppie-245 | Hess et al., 2014 |
| Ots_Prl2 | Hess et al., 2014 |
| Ots_RAD1104-38 | Hess et al., 2014 |
| Ots_RAD1832-39 | Hess et al., 2014 |
| Ots_RAD3513-49 | Hess et al., 2014 |
| Ots_RAD7936-50 | Hess et al., 2014 |
| Ots_RAD9480-51 | Hess et al., 2014 |
| Ots_RAS1 | Hess et al., 2014 |
| Ots_redd1-187 | Hess et al., 2014 |
| Ots_RFC2 | Hess et al., 2014 |
| Ots_SClkF2 | Hess et al., 2014 |
| Ots_SERPC1-209 | Hess et al., 2014 |
| Ots_SL | Hess et al., 2014 |
| Ots_slc7a2-71 | Hess et al., 2014 |
| Ots_stk6-516 | Hess et al., 2014 |
| Ots_TAPBP | Hess et al., 2014 |
| Ots_TCTA-58 | Hess et al., 2014 |
| Ots_TGFB | Hess et al., 2014 |
| Ots_Thio | Hess et al., 2014 |
| Ots_TLR3 | Hess et al., 2014 |
| Ots_TNF | Hess et al., 2014 |
| Ots_Tnsf | Hess et al., 2014 |
| Ots_tpx2-125 | Hess et al., 2014 |
| Ots_trnau1ap-86 | Hess et al., 2014 |
| Ots_txnip-321 | Hess et al., 2014 |
| Ots_u07-07.161 | Hess et al., 2014 |
| Ots_u07-17.135 | Hess et al., 2014 |
| Ots_u07-17.373 | Hess et al., 2014 |
| Ots_u07-18.378 | Hess et al., 2014 |
| Ots_u07-19.260 | Hess et al., 2014 |
| Ots_u07-20.332 | Hess et al., 2014 |
| Ots_u07-25.325 | Hess et al., 2014 |
| Ots_u07-49.290 | Hess et al., 2014 |
| Ots_u07-53.133 | Hess et al., 2014 |
| Ots_u07-57.120 | Hess et al., 2014 |
| Ots_u07-64.221 | Hess et al., 2014 |
| Ots_u1002-75 | Hess et al., 2014 |
| Ots_u1004-117 | Hess et al., 2014 |
| Ots_u1006-171 | Hess et al., 2014 |
| Ots_u1007-124 | Hess et al., 2014 |
| Ots_u1008-108 | Hess et al., 2014 |
| Ots_U211 | Hess et al., 2014 |
| Ots_U2362-227 | Hess et al., 2014 |
| Ots_U2362-330 | Hess et al., 2014 |
| Ots_U2446-123 | Hess et al., 2014 |
| Ots_U2567-104 | Hess et al., 2014 |
| Ots_U5049-250 | Hess et al., 2014 |
| Ots_U5121-34 | Hess et al., 2014 |
| Ots_UNKN4-150 | Hess et al., 2014 |
| Ots_UNKN6-187 | Hess et al., 2014 |
| Ots_USMG5-67 | Hess et al., 2014 |
| Ots_vatf-251 | Hess et al., 2014 |
| Ots_zn593-346 | Hess et al., 2014 |
| Ots_zP3b | Hess et al., 2014 |
| Ots_ZR-575 | Hess et al., 2014 |
| PGK-54 | Hess et al., 2014 |
| RAG3 | Hess et al., 2014 |
| S7-1 | Hess et al., 2014 |
| unkn526 | Hess et al., 2014 |
| Omm1080 | Seeb et al., 2007; Moran et al., 2013 |
| Ots201b | Seeb et al., 2007; Moran et al., 2013 |
| Ots213 | Seeb et al., 2007; Moran et al., 2013 |
| Ots9 | Seeb et al., 2007; Moran et al., 2013 |
| Ssa408uos | Seeb et al., 2007; Moran et al., 2013 |
